# Meta-analysis of drought-tolerant genotypes in *Oryza sativa*: A network-based approach

**DOI:** 10.1371/journal.pone.0216068

**Published:** 2019-05-06

**Authors:** Sanchari Sircar, Nita Parekh

**Affiliations:** Centre for Computational Natural Sciences and Bioinformatics, International Institute of Information Technology, Hyderabad, India; Louisiana State University, UNITED STATES

## Abstract

**Background:**

Drought is a severe environmental stress. It is estimated that about 50% of the world rice production is affected mainly by drought. Apart from conventional breeding strategies to develop drought-tolerant crops, innovative computational approaches may provide insights into the underlying molecular mechanisms of stress response and identify drought-responsive markers. Here we propose a network-based computational approach involving a meta-analytic study of seven drought-tolerant rice genotypes under drought stress.

**Results:**

Co-expression networks enable large-scale analysis of gene-pair associations and tightly coupled clusters that may represent coordinated biological processes. Considering differentially expressed genes in the co-expressed modules and supplementing external information such as resistance/tolerance QTLs, transcription factors, network-based topological measures, we identify and prioritize drought-adaptive co-expressed gene modules and potential candidate genes. Using the candidate genes that are well-represented across the datasets as ‘seed’ genes, two drought-specific protein-protein interaction networks (PPINs) are constructed with up- and down-regulated genes. Cluster analysis of the up-regulated PPIN revealed ABA signalling pathway as a central process in drought response with a probable crosstalk with energy metabolic processes. Tightly coupled gene clusters representing up-regulation of core cellular respiratory processes and enhanced degradation of branched chain amino acids and cell wall metabolism are identified. Cluster analysis of down-regulated PPIN provides a snapshot of major processes associated with photosynthesis, growth, development and protein synthesis, most of which are shut down during drought. Differential regulation of phytohormones, e.g., jasmonic acid, cell wall metabolism, signalling and posttranslational modifications associated with biotic stress are elucidated. Functional characterization of topologically important, drought-responsive uncharacterized genes that may play a role in important processes such as ABA signalling, calcium signalling, photosynthesis and cell wall metabolism is discussed. Further transgenic studies on these genes may help in elucidating their biological role under stress conditions.

**Conclusion:**

Currently, a large number of resources for rice functional genomics exist which are mostly underutilized by the scientific community. In this study, a computational approach integrating information from various resources such as gene co-expression networks, protein-protein interactions and pathway-level information is proposed to provide a systems-level view of complex drought-responsive processes across the drought-tolerant genotypes.

## Background

Drought is an inevitable consequence of drastic climate change. A look at the World Drought Map for the year 2017 from the Global Drought Information System [1] reveals the severity and intensity of drought across the globe. At this juncture, meeting the nutritional demands for an ever-increasing population is a challenge for policy makers. Although drought and heat waves have destroyed nearly a tenth of the cereal harvests, populations across the world continue to depend on them for food.

Irrigated rice occupies half the world’s rice field and accounts for ~75% of the global rice supply. The yield of this water-intensive crop is highly susceptible to climate change. Abiotic stress conditions like drought induces a wide array of physiological, morphological and molecular changes which might provide cues towards drought-responsive/adaptive mechanisms. However, these approaches are limited by the fact that nearly ~50% of the rice genes lack functional annotation for biological processes [2]. Analysis of high-throughput data (e.g., microarrays, RNASeq, proteomics, etc.) by computational methods such as network-based approaches, comparative genomics, etc. can be effective in functional characterization of rice genes, reducing the search space for identification of novel candidate genes and elucidating biological processes under various environmental conditions. Various drought tolerant rice genotypes exist, such as upland *indica* variety Bala with a high stomatal sensitivity to water loss and upland *japonica* variety Azucena with thick and deep roots. The deep-rooted and stress tolerant *aus* rice cultivar Nagina 22 (N22) is known to have enhanced enzymatic activity, α-linolenic acid metabolism and heat shock proteins [3,4]. Studies performed with indigenous cultivar Dagad Deshi [5] also identified quantitative traits associated with grain yield. The drought tolerant upland variety IRAT109 is known for its superior yield and other parameters compared to the lowland Zhenshan97 under drought stress [6] and have been used for studying traits like osmotic potential [7]. Thus there has been a continuous effort in conventional breeding strategies to develop high-yielding drought-tolerant rice varieties.

Currently, a large number of gene-expression data corresponding to drought tolerant genotypes are available in public domain. Systematic meta-analysis of these datasets can help in increasing the reproducibility and provide new biological insights. Numerous methods are available to merge gene expression data and remove batch effects, *viz*., Z-score standardization, mean centering [8], cross-platform normalization [9], empirical Bayes method for smaller batch size [10], etc. Network-based approaches help in summarizing large-scale data with thousands of genes into manageable clusters or functional modules. Further, these approaches help to prioritize important genes based on their topological parameters such as degree, betweenness and eigen-gene centralities, apart from fold-change and functional enrichment. For example, co-expression networks are popularly used to draw relationship between genes and identify tightly coordinated biological processes and functional characterization of novel genes [11–15]. An integrative approach combining co-expression networks with protein-protein interactions and metabolic pathway information can help in associating genotype to phenotype to a considerable extent [16–18].

In this study, we carried out meta-analysis of drought-tolerant rice genotypes under drought stress using network-based approach with the objective of identifying gene signatures and processes that are hallmark of drought tolerant species. For this analysis, from the six microarray datasets containing seven drought-tolerant genotypes (Azucena, Bala, Nagina, Dagad deshi, Vandana, IRAT109 and *Oryza* DK151), 57 samples corresponding to the shoot tissue and seedlings from whole plant (Affymetrix platform) are considered (Table 1). Samples corresponding to drought-sensitive genotypes or root tissue in these datasets are not considered for the analysis (see S1 Table for dataset details). The R package, Weighted Gene Co-expression Network Analysis (WGCNA), is used to capture interaction patterns between genes [19] resulting in co-expressed gene modules. These co-expressed modules are associated with additional information such as enrichment of differentially expressed genes, transcription factors, known stress/tolerance associated QTLs, module-trait associations and gene ontology, to identify drought-responsive modules. The key driver genes in these modules are identified using topological network parameters and up- and down-regulated protein-protein interaction networks are constructed from known interaction of these genes. Tightly coupled gene clusters that may be associated to various signalling and metabolic processes likely to be affected due to drought are analyzed.

**Table 1:** Drought-tolerant samples from Affymetrix datasets from NCBI-GEO and ArrayExpress considered for the meta-analytic are listed.

Microarray studies (Total No. of Samples)	Title of the Experimental Studies	No. of Control + Drought Samples Considered	Tissue Samples Considered	RT-PCR validation by individual studies
GSE41647* (18)	Transcriptome profiling for drought tolerant and susceptible cultivars of Indica rice. (**PMID: 28181537**)	9 Dagad deshi (DD)	Seedlings (whole plant)	Yes
E-MEXP-2401* (12)	Transcription profiling of Oryza sativa subtypes Cultivar Nagina-22 (N22) and IR64 subtypes under normal and drought conditions (**PMID:20809928**)	6 Nagina22 (N22)	Seedlings (whole plant)	Yes
GSE21651* (16)	Differential expression for salt and drought stress from tolerant and sensitive lines (**PMID: 29785071**)	4 Vandana	Seedlings (leaf)	Yes
**GSE26280**^#^ **(36)**	Genome-wide temporal-spatial gene expression profiling of drought responsiveness in rice (**PMID: 21406116**)	6+6+6 *Oryza* DK151	Leaves (tillering + panicle elongation + booting) (vegetative + reproductive stages)	Yes
**GSE24048** **(12)**	Expression data from field droughted rice plants	12 6 Bala + 6 Azucena	Leaves (vegetative stage)	Unpublished
GSE25176* (16)	Expression data from rice varieties IRAT109 (resistant) and ZS97 (sensitive) for drought stress treatment in flag leaves (**PMID: 23459234**)	8 IRAT109	Flag leaf (reproductive stage)	Yes

*These datasets also have drought-sensitive samples which are not used in this study

^#^ This dataset had root tissue samples from tillering and panicle elongation stages and young panicle tissue in booting stage which are not used in this study.

## Methods

### Datasets

For this analysis, six microarray datasets containing seven drought-tolerant genotypes (Azucena, Bala, Nagina, Dagad deshi, Vandana, IRAT109 and Oryza DK151), corresponding to Affymetrix Rice Genome Array from NCBI (platform accession: GPL2025) and the EBI ArrayExpress Archive are considered. The details of the datasets are summarized in Table 1. The validation of gene expression by real-time PCR has been reported for five out of six studies and shown to exhibit good correlation with the microarray data generated. The conditions, stages and tissues are given as follows:

**GSE41647**: Hydroponically grown 7-day-old seedlings of Dagad deshi, subjected to drought stress by placing them on 3 mm Whatmann sheets under light for 3 h and 6 h at 28±1°C. For control samples, seedlings were kept in RGM (root growth medium) for 6 h at 28±1°C.
**E-MEXP-2401**: Seedlings from Nagina-22 (N-22, tolerant) were grown side by side in plastic pots for 14 days at 28±1°C with a daily photoperiodic cycle of 14/10 h light/dark provided by fluorescent tubes. Sterile absorbent cotton soaked with Hoagland’s solution was used as seed bed for growing rice seedlings. Water stress was given to the plants by withholding water supply till visible leaf rolling appeared in the plants.
**GSE21651**: For drought stress, seeds of Vandana were sown in Hoaglands nutrient solution. After 14 days seedlings were blot dried and kept in air for 12 hrs and leaf samples were collected for RNA extraction.
**GSE26280**: Drought tolerant rice line, DK151 (an F7 line derived from a cross between two DT IR64 introgression lines (ILs), DGI 187 and DGI 74) were used gene expression patterns across leaves and roots at tillering stage and panicle elongation stage, leaves and young panicle at booting stage. Stress at three different stages were applied: 4-tiller (tillering) stage, panicle elongation stage, and booting stage. Plants were stressed until the leaf became fully rolled at noon (RWC: 65%-75%). It took three and two days for drought stress to become apparent at the tillering and panicle elongation stages and at booting stage, respectively. Both leaf and root samples were collected for the first two stages, and leaves and young panicle samples were collected at the booting stage. In the current study, only leaf tissue was considered.
**GSE24048**: two drought tolerant cultivars, Bala and Azucena were grown in 1.2 m2 plots under flooded conditions. Starting at 59 days after sowing, drought was imposed by withholding water, while a set of control plots had continued flooding conditions. The drought was imposed for 24 days during which time a small amount of water was added on 3 occasions to raise soil moisture to 30% by volume. After 24 days the second youngest fully expanded leaf was taken and gene expression analysis performed.
**GSE25176**: IRAT109 (drought-resistant) were grown in PVC pipes. For half of the plants, two drought-rehydration cycles were performed to each plant in PVC pipes at the booting stage (about 14 days before flowering). When all leaves of a stressed rice plant became completely rolled, watering was applied to the full capacity of the pipe, and the second cycle of drought stress was applied until all leaves became completely rolled again and watering was resumed for the rest of the lifecycle. Four samples with RWC in the range of 94%–95% (no stress, D0), 83%–88% (slight drought in which leaves were slightly rolled, D1), 74%–78% (moderate drought in which about half of each leaf was rolled, D2) and 65%–69% (severe drought in which all leaves were completely rolled, D3) were collected for expression profiling analysis. For the current study, DEGs at D3 *vs*. D0 were considered.

### Data Pre-processing

A first step in any transcriptomic studies is to check the data quality. All the samples were found to pass quality check on using the package, ArrayQualityMetrics that assesses reproducibility, outlier arrays, batch effects and computes measures of signal-to-noise ratio [20]. Datasets were normalized using Robust Multiarray Averaging (RMA) package to remove systematic variations due to different experimental conditions, dye effects, uneven hybridizations, etc. [21]. In a meta-analytic approach, when multiple datasets from different experimental conditions are combined, batch-specific variations need to be eliminated. For this, COMBAT, an Empirical Bayes method in the R package inSilicoMerging, was used to merge the expression values across the datasets [10]. Probes having no gene annotations, mapping to more than one gene annotation (ambiguous probes), or having very low intensity values (< 20 across all samples) were removed. For multiple probes mapping to the same gene, the one exhibiting a higher coefficient of variation across the samples was considered. This resulted in 14,270 genes that were used for the construction of co-expression network.

### Co-expression network construction

The weighted gene correlation network analysis (WGCNA) package was used to construct a ‘signed’ co-expression network of drought tolerant rice samples from normalized, log2-transformed expression matrix across 14,270 genes and 56 samples (one sample, GSM645335 was detected as an outlier on performing sample-wise clustering in WGCNA and removed). The unsigned networks use absolute value of correlations, $s_{unsigned}^{ij}=|cor(x_{i},y_{j})|$, and are unable to distinguish between gene activation (s^ij^_unsigned_ = 1) and gene repression (s^ij^_unsigned_ = 1), leading to loss of biological information [22]. Hence, here we construct a signed co-expression network, taking into account the ‘sign’ of correlation between expression profiles of genes and the similarity measure in this case is defined as:

$$s_{signed}^{ij}=\frac{\left(1+cor(x_{i},y_{j})\right)}{2},$$

where *x*_*i*_ and *x*_*j*_ are the expression profiles of genes *i* and *j* across the microarray samples. Here, s^ij^_signed_ = 1 corresponds to positive correlation, s^ij^_signed_ = 0, negative correlation and s^ij^_signed_ = 0.5, no correlation, thereby distinguishing between positively and negatively correlated genes. The similarity between gene-pairs is computed using signed Pearson’s correlation matrix, scaled to power β = 18 (approximates scale free-topology criterion), and the parameters of the signed co-expression constructed are summarized in Table 2. The function *block-wiseModules* was used for hierarchical clustering of genes using Dynamic Tree Cut approach [23] with maximum block size = 15000, minimum module size = 100, “cut height” = 0.995 and “deep split” = 2. This resulted in 13 co-expressed gene modules ranging in size from 2686 (turquoise) to 250 (salmon) genes, and 1431 genes are left unclustered (in grey module). The co-expression network with all the annotations are submitted as S2 Table.

**Table 2:** Parameters for the construction of signed, weighted gene co-expression network is summarized.

Genotype	No. of Samples	No. of Genes	β Cutoff	R^2^ Scale-free fit	Mean k	Median k	Max k	No. of Modules
Drought Tolerant	56	14270	18	0.85	53.4	30.9	331	13

### Statistical and biological significance of network modules

#### Statistical significance of network modules

To assess the statistical robustness of the co-expressed modules, module quality statistics were computed by re-sampling the dataset using the *modulePreservation* function in WGCNA. It randomly permutes the gene labels 200 times and computes various network quality statistics such as density, module membership, connectivity, etc. for each module. The log *p*-values and *Z*-scores for each module are summarized as *psummary* and *Zsummary* (see S3 Table). The *Z*-score provides evidence that a module is preserved more significantly than a random sampling of genes and *p*-value gives the probability of seeing the module quality statistic in a random sampling of genes of the same size. It is observed that the *psummary* value is very low (∼ 0.0) and *Zsummary* > 10, providing strong evidence of network connectivity preservation and robustness of the co-expressed modules [24].

#### Biological relevance of network modules

Gene ontology and enrichment analysis of the modules carried out using agriGO [25] indicates probable pathways captured by the module-genes. After merging the six datasets using inSilicoMerging, differentially expressed genes (DEGs) were identified using the criteria of average fold-change across 6 datasets ≥ |1.2| and a *t*-test with *p*-value ≤ 0.05. A total of 6441 DEGs were identified and mapped on to the co-expressed modules (Table 3). We observe a clear demarcation in the distribution of up- and down-regulated genes on the modules, clearly showing the effect of constructing a ‘signed’ network. The Turquoise, Yellow, Tan and Brown modules are enriched with only up-regulated genes (3012) while Blue, Green, Red, Magenta, Salmon and Purple modules contain mainly down-regulated genes (3235) and these modules are likely to capture processes that are activated or repressed in response to drought, respectively.

**Table 3:** Co-expressed modules with percentage DEGs, transcription factors (TFs) and GO enriched terms are shown. Modules are ordered by their size.

Module	Size	DEGs (%age)		TFs (from PlnTFDB)		GO Analysis using agriGO
		Up	Down	Up	Down	
Turquoise	2686	2103 (78.3)	0	171	0	regulation of cellular process (204), catabolic process (63), transcription (165), localization (160)
Blue	2500	0	1861 (74.4)	0	99	small molecule metabolic process (156), photosynthesis (36), cellular nitrogen compound metabolic process (60), oxoacid metabolic process (78), establishment of localization (179)
Brown	1327	247 (18.6)	0	7	0	RNA processing (25), gene expression (119), DNA repair (17), lipoprotein metabolic process (7)
Yellow	1203	561 (46.6)	3 (0.25)	43	0	Transport (110), catabolic process (42), establishment of localization (104), small molecule metabolic process (70), generation of precursor metabolites and energy (31)
Green	1192	0	713 (60)	0	57	protein modification process (121), post-translational protein modification (112), phosphate metabolic process (105)
Red	884	9 (1)	286 (32.3)	1	4	ncRNA metabolic process (43), RNA processing (41), translation (67), gene expression (124), RNA modification (19), ribosome biogenesis (20)
Black	677	10 (1.5)	20 (3)	2	4	protein modification process (73), transport (62), macromolecule modification (73), signal transduction (21)
Pink	633	42 (6.6)	22 (3.5)	0	1	establishment of localization in cell (38), intracellular transport (34), vesicle-mediated transport (30)
Magenta	415	0	187 (45)	0	8	cellular glucan metabolic process (10), polysaccharide metabolic process (11), cellulose biosynthetic process (6)
Purple	390	0	95 (24.4)	0	6	cellular protein metabolic process (48)
GreenYellow	347	2 (0.6)	86 (24.8)	0	3	Translation (75), gene expression (92), cellular protein metabolic process (85), cellular biosynthetic process (103)
Tan	335	101 (30)	0	8	0	-
Salmon	250	0	93 (37.2)	0	9	gene expression (35), regulation of metabolic process (24), regulation of transcription (22)

### Construction of minimal drought-adaptive PPI networks

The modules obtained in the co-expression network are in general quite large and as such are not very useful. Also, fold-change alone does not say much about the systems-level significance of a gene. To address some of these issues, we additionally consider network topological measures such as intra-modular connectivity/degree centrality (k_IM_), which help in identifying ‘hub’ genes and eigengene-based centrality (k_ME_), which is a measure of module membership of a gene obtained by correlating its expression profile with eigengene of the corresponding module, to filter important stress-responsive genes. Top 20% genes ranked based on high k_IM_ and k_ME_ values that are also differentially expressed (fold-change ≥ |1.2|) were selected from each of the 4 up-regulated (Turquoise, Yellow, Tan and Brown) and 6 down-regulated (Blue, Green, Red, Magenta, Purple and Salmon) drought-responsive modules. This resulted in 754 up-regulated and 893 down-regulated genes. These stress-responsive genes were used as ‘seeds’ to search the database of protein-protein interaction networks, STRING [26] and a total of 939 interactions between 425 up-regulated genes and 8639 interactions between 640 down-regulated genes were identified. The remaining unmapped genes were queried in co-function networks, RiceNet [27] and their orthologs in AraNet [28], and additional interactions (between 76 up-regulated and 80 down-regulated genes respectively) that were conserved between both RiceNet and AraNet were retrieved. This resulted in two PPINs which we refer to as the up-regulated drought tolerant network (uDTN) consisting of 466 nodes and 1015 edges, and the down-regulated drought tolerant network (dDTN), consisting of 665 nodes and 8719 edges. The largest connected component in these two PPINs was considered for further analysis. The largest connected component of uDTN containing 422 nodes and 990 edges, and that of dDTN with 623 nodes and 8627 edges, was observed to follow an approximate power law degree distribution (with R^2^ value of 0.85 and 0.76 respectively), indicating scale-invariant topology. These two components were then subjected to Markov Cluster Algorithm (MCL). Tightly coupled gene clusters (13 in uDTN and 14 in dDTN) of size ≥ 5 and showing significant functional enrichment in terms of pathway, GO or domain in STRING database were identified. We expect these gene clusters to provide insight into the biological processes that are activated or repressed in response to drought stress in drought tolerant varieties. The networks were visualized using Cytoscape [29] and topological analysis is carried out using NetworkAnalyzer plugin.

## Results

### Identification of drought-adaptive modules

The signed weighted co-expression network of 14,270 genes resulted in 13 co-expressed modules. These modules are large, comprising hundreds and thousands of genes. To identify key drought-responsive processes we carry out various statistical operations discussed below.

### Differential gene expression analysis and GO enrichment

GO enrichment analysis of the 13 co-expressed gene modules is carried out using agriGO [25] and the top enriched GO terms are given in Table 3. The number of up- and down-regulated genes (DEGs) and known rice transcription factors (TFs) extracted from PlnTFDB [30] are identified and mapped on these modules. The results are summarized in Table 3. The DEGs are identified based on fold-change (≥ |1.2|) and filtered using *t*-test with *p*-value (≤ 0.05). It is evident from Table 3 that Turquoise module has the highest number of up-regulated genes (2103). It harbors genes involved in the regulation of various cellular processes and transcription factors such as bZIPs, NACs and MYBs, which are known to be involved in drought stress. The next major up-regulated module is the Yellow module with 561 DEGs. Apart from being involved in transport and localization, this module also consists of genes involved in catabolic processes and energy generating processes. Blue module has a large number of down-regulated genes (1861), majority of which are involved in photosynthesis and associated metabolic processes known to be down-regulated in shoot tissue during drought [31,32]. Green and Magenta modules also contain a significant fraction of down-regulated genes (713 and 187 respectively) and are involved in protein synthesis and cellulose metabolism respectively. Both these processes are known to be hampered during drought as a result of growth retardation and damage to membranes [33]. The Red module contains 286 down-regulated genes involved in gene expression, ribosome biogenesis and RNA processing, indicating a finer regulatory mechanism controlling protein synthesis. Based on GO enrichment analysis and distribution of DEGs, we associate 4 modules (Turquoise, Yellow, Brown and Tan) with up-regulated processes and 6 modules (Blue, Green, Red, Magenta, Purple and Salmon) with down- regulated processes.

Transcription factors play an important role in translating stress-induced signals to cellular responses by binding to specific *cis*-elements of downstream target genes. A number of regulons, *viz*., like AREB/ABF regulon, DREB1/DREB2 regulon, NAC regulon, MYB and WRKY TFs have been identified to play an important role in stress response. Interactions between the regulons such as AREB/ABFs and NACs have been reported where NAC TFs regulate ABA biosynthetic genes. NAC-induced regulation of ABA regulons and presence of ABRE elements in the promoter regions of SNAC TFs hints at complex cross talks between the regulons [34–36]. Similarly, MYB TFs have been reported to be involved in stomatal patterning, regulation of guard cells controlling stomatal movements indicating an overlap with the ABA signals [37–39]. The DREB genes have been shown to physically interact with AREB/ABFs [40]. We show that our co-expressed modules are able to capture this inter-dependence of TFs and genes shown to be involved in drought tolerance.

To identify the differentially expressed TFs associated with the regulons, we analyzed their distribution across the 13 co-expressed modules. For this analysis, known rice transcription factors (2385 genes) are downloaded from PlnTFDB (3.0) [30]. Of the 776 TFs that mapped to the co-expression network, 232 are up-regulated and 192 down-regulated. The distribution of these differentially expressed TFs on the co-expressed modules is summarized in Table 3. We observe that 171 of the up-regulated TFs mapped to Turquoise, 43 to Yellow, and 8 and 7 to Tan and Brown modules respectively, while 99 of the down-regulated TFs mapped to Blue, 57 to Green, 9 to Salmon, 8 to Magenta, etc.

**Fig 1:**
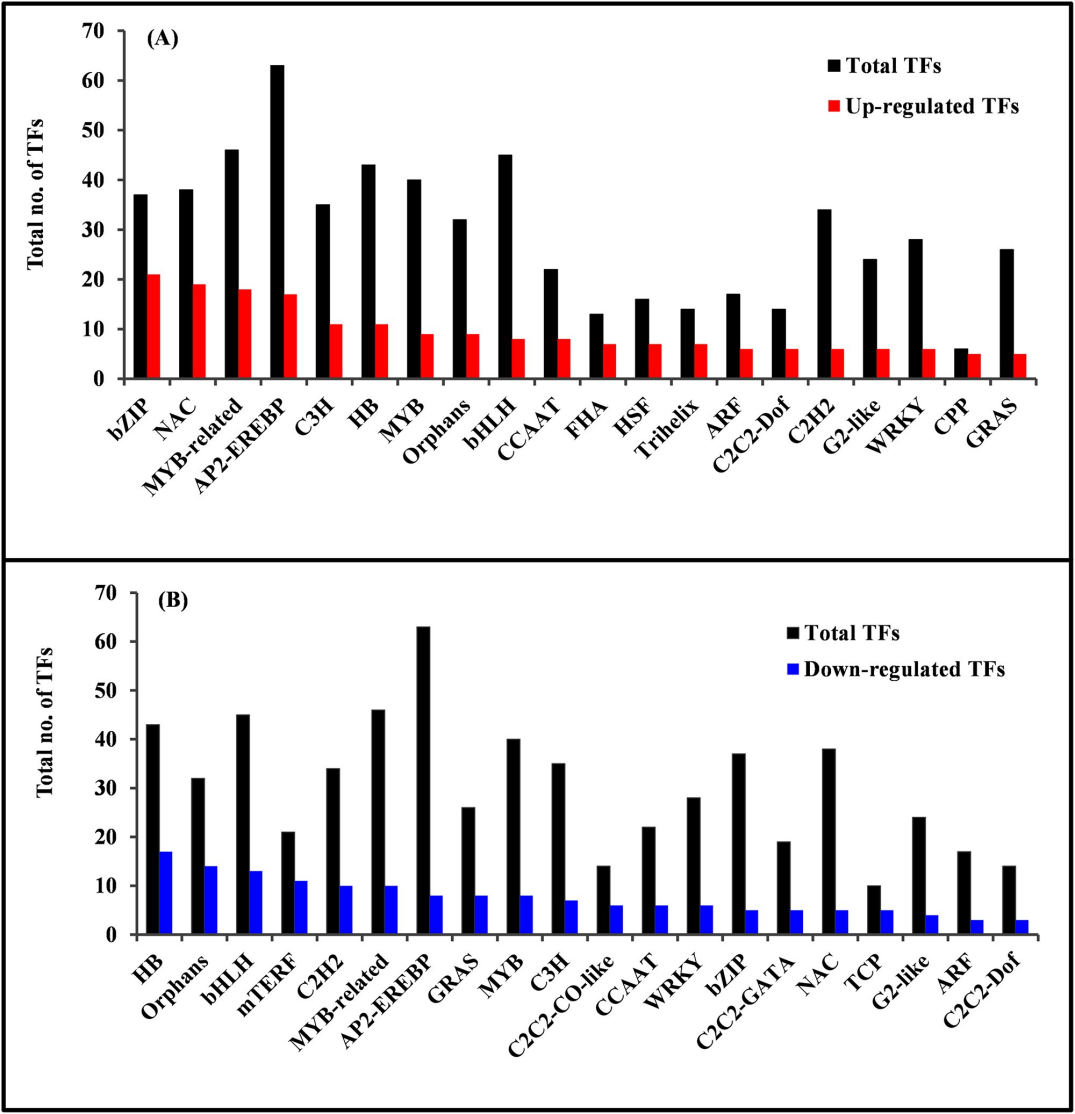
Transcription factor (TF) families identified in our co-expression network are ranked based on number of differentially expressed members (fold-change ≥ |1.2| and p-value ≤ 0.05). In (A) top 20 up-regulated TF families (‘red’ bars), and (B) top 20 down-regulated TF families (‘blue’ bars) are shown. The vertical bars in ‘black’ depict the total number of members of the respective TF families in (A) and (B). The gene-ID-TF mapping is taken from PlnTFDB v3.0.

In Fig 1(A) and 1(B), the total number of known members of various TF families that mapped to our co-expression network is depicted in ‘black’, while the ‘red’ and ‘blue’ bars depict the number of known up- and down-regulated TFs respectively. It is evident from Fig 1(A) that high numbers of up-regulated TFs belong to bZIP family followed by NAC, MYB-related and AP2-EREBP TFs, while from Fig 1(B) we observe that high numbers of HB, Orphans and bHLH are down-regulated. In plants, bZIP TFs regulate diverse processes including seed germination, flowering, photomorphogenesis, abiotic stress and ABA signalling [41]. Among the 21 up-regulated bZIP TFs, 17 are mapped to Turquoise module, including the well characterized OsABF1 (OsABI5/OREB1/OsbZIP10) TF. Recent study indicates that OsABF1 is a universal positive regulator of drought tolerance and ABA signalling in rice. It directly regulates OsbZIP23, OsbZIP46, and OsbZIP72 all of which are a part of Turquoise module [42]. It is also noted that both OsABF1 and OsbZIP23 are ‘hubs’ in the module (top 20% high degree genes). The NAC regulon plays an important role in stress response under various abiotic and biotic stress conditions as well as in various developmental programs such as lateral root development, flower development, formation of secondary walls, maintenance of secondary walls, etc. [43]. Among the 19 up-regulated NAC TFs, 14 mapped to Turquoise module, 4 to Yellow module and 1 to Brown module. For example, OsNAC52 which has been shown to be involved in drought tolerance [44] and LOC_Os07g48450 (yet to be characterized in drought stress) are observed to have a significant fold-change and high degree in the Turquoise module. The MYB-type TFs are one of the most abundant TFs in plants and play essential roles in development, growth and stress response and have been classified based on the number of MYB repeats [45]. We observe 18 MYB-related and 9 MYB TFs that are up-regulated, many of which are not yet functionally characterized for stress tolerance except for OsMYB55, reported to be involved in heat tolerance and amino acid metabolism and OsMYB91, in plant growth and salt tolerance [46]. We also other 8 MYB and 10 MYB-related TFs to be down-regulated (Fig 1(B)). Among these, the MYB TF LOC_Os01g44390 (OsRAD1), involved in floral development, is significantly down-regulated and is also a ‘hub’ gene in the Blue module. The other class of important TFs are AP2-EREBPs which are highly abundant and involved in development, sugar signalling, ethylene response, pathogen response and abiotic stress [47,48]. We observe 17 up-regulated AP2-EREBPs TFs in our co-expression network, of which 13 map to Turquoise module and 2 each to Yellow and Tan modules. The DREB genes of this TF family bind to dehydration responsive element (DRE)/C-repeat (CRT) regions and activate LEAs, dehydrins, starch-degrading enzymes, Hsps, etc. [49] and play a role in stress response pathway. In Fig 1(B), we observe 17 homeobox genes to be down-regulated. These genes encode TFs which are important regulators for development, cell fate and body plan specification [46]. Majority of these TFs are part of the Blue module. Transcription factors (9 up-regulated and 14 down-regulated) belong to the Orphans category in PlnTFDB, which corresponds to TFs that could not be categorized into existing TF families. We observe 4 WRKY TFs in Green module, *viz*., WRKY45 (associated with disease resistance [50]), WRKY47 (positive regulator drought in *P*_*SARK*_::*IPT* plants [51]), WRKY68 and WRKY74, and WRKY69, WRKY109 part of Black and Blue module respectively. All these WRKYs are down-regulated.

Thus, we observe that the coordinated up-regulation of TFs corresponding to different regulons are part of the same modules (mainly Turquoise and Yellow), while TFs associated with disease resistance, flowering and development are part of the down-regulated (mainly Blue and Green) modules.

### QTL genes mapping to Co-expressed modules

To further validate the biological significance of the 10 co-expressed modules in response to drought stress, we mapped known quantitative trait locus (QTL) genes on these modules. For this analysis, genes spanning QTLs that are associated with resistance/tolerance traits are obtained from Q-TARO database [52] and mapped on to the co-expressed modules. We identified 249 QTL genes in our co-expression network that are reported to be associated with resistance/tolerance traits in Q-TARO database. Of these, 73 QTL genes are up-regulated, 67 down-regulated and 109 are not differentially expressed.

**Fig 2:**
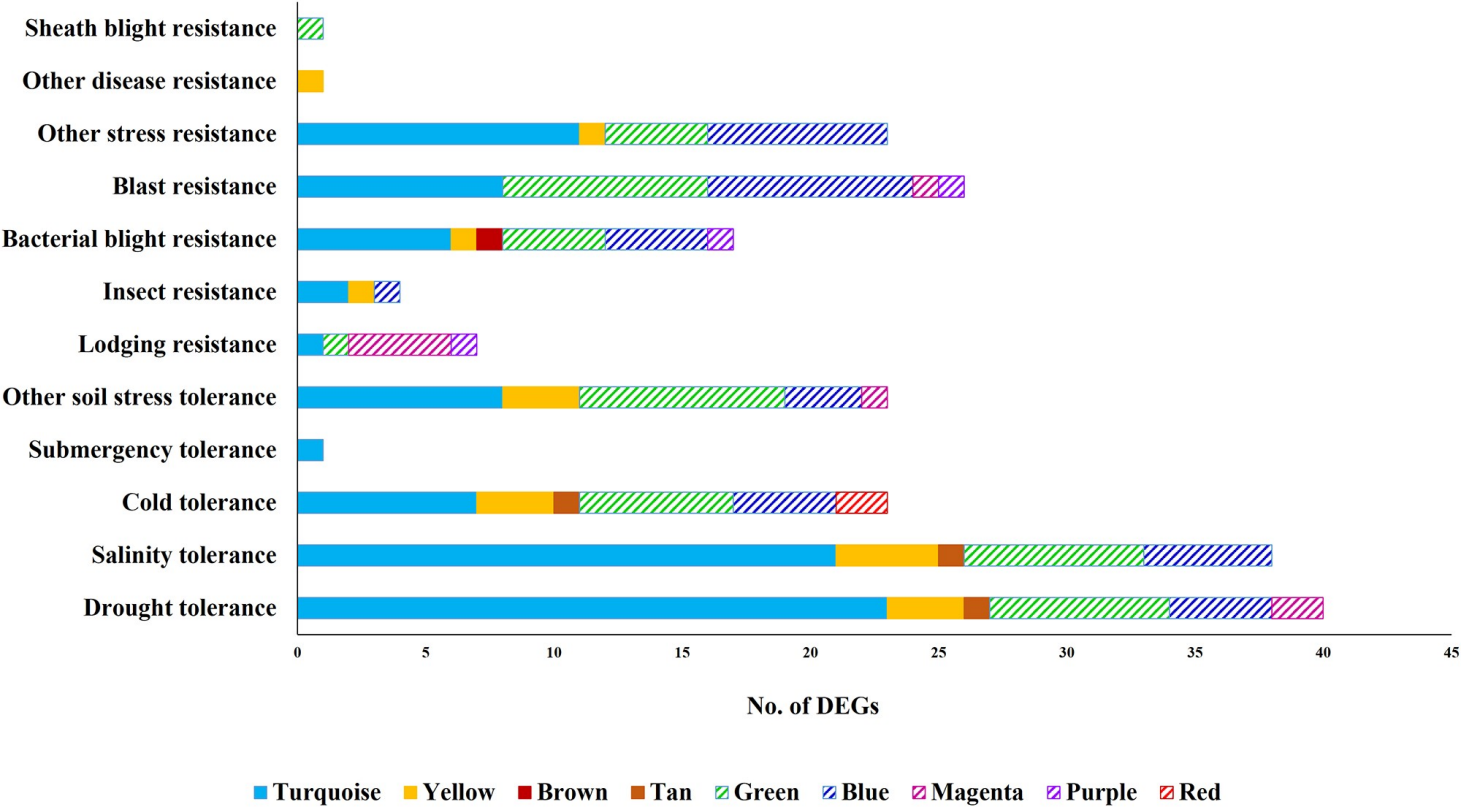
Number of differentially expressed genes (DEGs) from the co-expressed modules that span the QTLs associated with various stress conditions is depicted. The solid bars represent modules with up-regulated genes, while striped bars represent modules with down-regulated genes. It may be noted that a significant number of DEGs from Turquoise, Yellow, Green and Blue modules are associated with osmotic stress (drought and salinity tolerance). The gene ID-QTL mapping is taken from the Q-TARO database.

As shown in Fig 2, twenty seven of the 73 up-regulated genes are associated with drought tolerance and mapped to the up-regulated modules, Turquoise (23 genes), Yellow (3) and Tan (1), while thirteen of the 67 down-regulated genes are associated with drought and mapped to the down-regulated modules, Green (7), Blue (4) and Magenta (2). Some of these drought tolerance QTL genes that mapped to the Turquoise module are known transcription factors, *viz*., OsHOX22, bZIP TFs (e.g., OsABF1, OsbZIP23 and ABI5-Like1(ABL1)), and NAC TFs (e.g., OsNAC6 and OsNAC2). Apart from these QTL genes, Turquoise module also harbors QTL genes associated with salinity (21), cold stress (7), other soil stress tolerance (8), blast resistance (8), bacterial blight (6), etc. This indicates common stress responsive mechanisms in abiotic and biotic stress conditions. Previous studies suggest that drought and salinity responses share common pathway components and regulators in the plant [53]. This seems to be well captured in our study with bZIP TFs involved in ABA-mediated stress response under drought stress, kinases (SnRK2-type SAPK4) with a role in ABA signalling and also implicated in the regulation of ion homeostasis and salinity tolerance [54], PLDα involved in ABA-regulated stomatal closure, zinc-finger proteins (e.g., ZOS3-21—C2H) involved in salt tolerance and ABA induced anti-oxidant defense, being up-regulated and co-expressed together in the same module. We also observe some of the QTL genes associated with biotic stress to be up-regulated and co-expressed with drought-responsive genes in Turquoise module. For example, selenium-binding gene OsSBP (associated with increased H_2_O_2_), OsGH3.2 involved in modulating auxin and ABA as well as in pathogen defense by suppressing pathogen-induced IAA accumulation, OsWRKY31 (involved in auxin signalling) and OsNAC6 TFs (involved in broad spectrum of resistance including wounding and blast disease). Similarly, majority of the down-regulated genes associated with blast resistance such as the well characterized WRKY45 and OsNPRI involved in salicylic acid (SA) signalling pathway and defense response, some drought-associated QTLs, e.g., OsGSK1 associated with brassinosteroid (BR)-signalling and flowering, three AP2-EREBP TFs (OsDREB1E, OsDREB6, OsDREB1B) involved in ethylene singling are down-regulated and part of the Green module.

### Analysis of minimal drought-adaptive modules

To understand the core drought-responsive signalling and metabolic changes, we discuss below the analysis of two PPI networks, uDTN (comprising 466 genes and 1015 interactions) and dDTN (comprising 665 nodes and 8719 interactions). These networks integrate protein-protein interactions with co-expressed gene profiles for the DEGs that are topologically significant (top 20% k_IM_ and k_ME_ values) in the co-expression network. As discussed in Section 1.4, protein-protein interactions for these gene sets are extracted from STRING database and co-function networks, RiceNet and AraNet (S4 and S5 Tables).

### Analysis of uDTN

To identify the processes that are induced in response to drought stress, we applied MCL algorithm to identify tightly coupled gene clusters of genes (size ≥ 5) that are functionally enriched in STRING database for domains, pathways, or GO terms. This resulted in 13 clusters summarized in Table 4. Among the clusters shown here with a total of 151 genes, 83 edges have experimental evidences from model organisms (confidence score ≥ 0.4 in STRING DB). A brief description of the biological processes captured by these clusters is given below based on annotations in MapMan [55] and RGAP [56].

**Table 4:** Functional enrichment of gene clusters identified in up-regulated drought tolerant network (uDTN) using MCL algorithm. (TQ: Turquoise, Y: Yellow, BR: Brown, T: Tan).

Cluster	Associated Biological Processes	Total Genes	Cluster	Associated Biological Processes	Total Genes
**U1**	ABA-signalling and secondary metabolism	35 (23TQ, 10 Y, 2BR)	**U8**	Ubiquitination	8 (4TQ, 4Y)
**U2**	Molecular chaperons and heat stress TFs (Hsfs)	19 (8TQ, 2T, 9Y)	**U9**	TCA cycle	6 (5Y, 1BR)
**U3**	Cell wall and amino acid metabolism	16 (13TQ, 3Y)	**U10**	Protein kinases	5 TQ
**U4**	Amino acid degradation and mitochondrial ETC	15 (13TQ, 2Y, 1T)	**U11**	Mitochondrial ETC/ATP synthesis	5 (3Y, 2TQ)
**U5**	RNA binding and processing	13 6BR, 4TQ, 2T, 1Y)	**U12**	Starch synthesis and degradation	5 TQ
**U6**	Protein degradation	10 (5Y, 5TQ)	**U13**	bZIP TFs	5 TQ
**U7**	Plant defence system	9 (7TQ, 1Y, 1BR)			

### Cluster U1, U10 and U13: Components of ABA signalling, primary metabolism and biosynthesis of secondary metabolism

**Fig 3:**
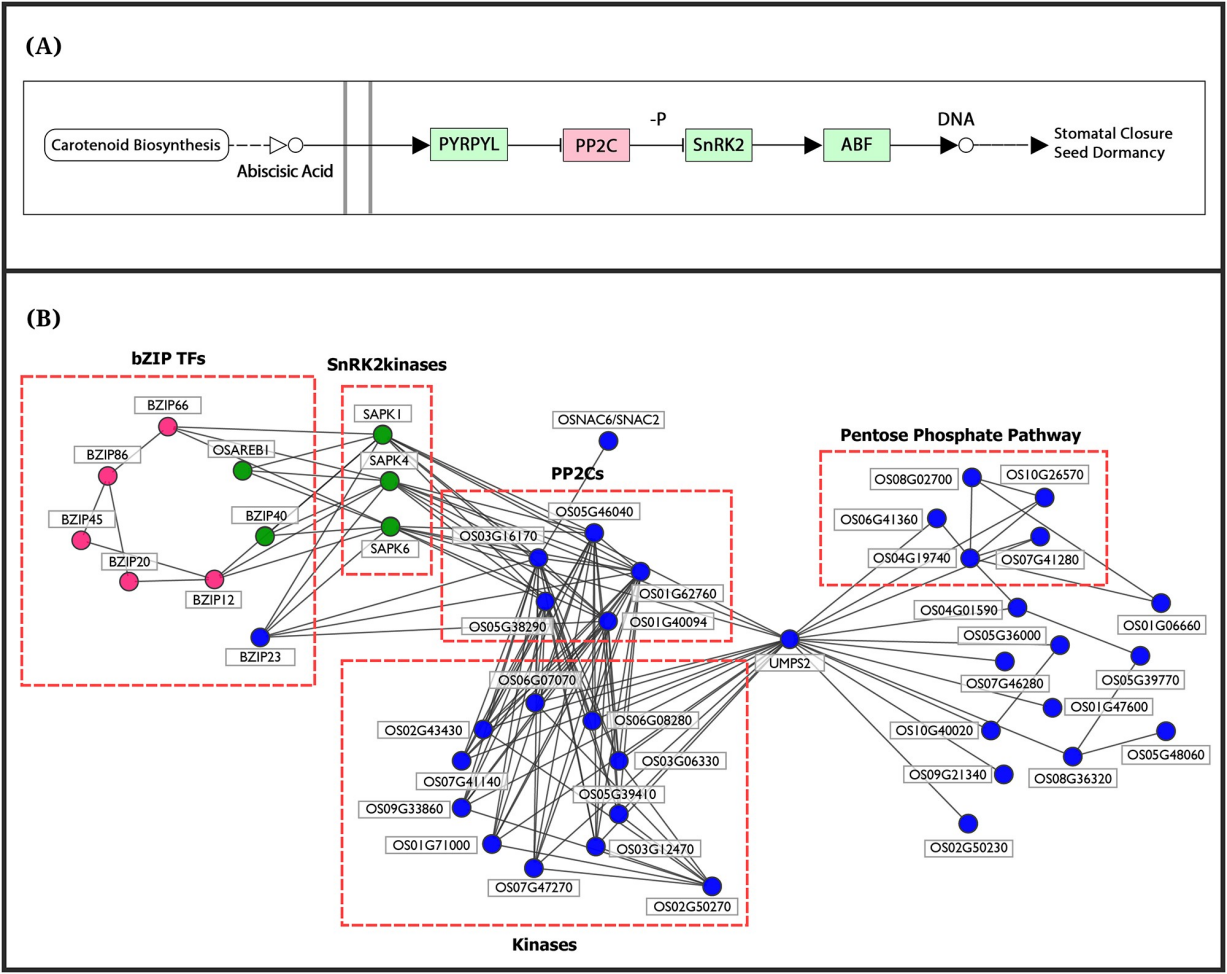
(A) Components of ABA signalosome complex: PYR/PYL receptors, PP2Cs, SnRK2 kinases and ABF/bZIP transcription factors are depicted (reproduced from KEGG). (B) Subnetwork of Cluster U1 (‘blue’), Cluster U10 (‘green’) and Cluster U13 (‘pink’) obtained using MCL algorithm on up-regulated drought-tolerant network (uDTN) is seen to capture the crosstalk between the signalling components: PP2Cs, kinases, bZIP23, OsNAC6, SnRK2 kinases (Cluster U1), bZIPs (Cluster U1 and Cluster U13) with metabolic components of Cluster U1 via UMPS2. This clearly indicates the role of UMPS2 as a novel candidate for drought.

From our analysis, we observe that the three clusters, U1, U10, and U13, capture the abscisic acid (ABA) signal transduction process, and components of primary and secondary metabolism. It is known that on perceiving abiotic stress, the adaptive response of plants is controlled mainly by the phytohormone, ABA. Under drought stress, a sudden increase in ABA synthesis is induced accompanied by various physiological and morphological changes in the plant, namely, regulation of growth, stomatal closure, hydraulic conductivity, etc. However, mechanisms of fine-tuning the ABA-levels are not yet well understood. Earlier studies suggest that stress responsive genes mediate through at least two pathways: ABA-dependent and ABA-independent, with a possible crosstalk between their components. Calcium, which serves as a second messenger for various stresses, is proposed to be a strong candidate, mediating such cross talks. Recent progress in our understanding of ABA signal transduction indicates that the central signalling module comprises three protein classes: Pyracbactin Resistance/Pyracbactin resistance-like/Regulatory Component of ABA Receptor (PYR/PYL/RCARs) proposed to be the ABA receptors, Protein Phosphatase 2Cs (PP2Cs) which act as negative regulators, and SNF1-related protein kinases 2 (SnRKs) which are positive regulators. In the presence of ABA, the PYR/PYL/RCAR-PP2C complex formation leads to inhibition of PP2C activity, allowing activation of SnRKs which target membrane proteins, ion channels and transcription factors, and facilitate transcription of ABA-responsive genes (see Fig 3(A), reproduced from KEGG [57].

We observe that the subnetwork comprising three gene clusters in Fig 3(B): U1 (35 genes in blue), U10 (5 genes in green) and U13 (5 genes in pink) capture very well essential components of the ABA-signalling pathway (Fig 3(A)). Based on functional analysis, U1-cluster genes can be divided into two groups, one involved in signalling processes and the other involved in primary metabolism and biosynthesis of secondary metabolites. The genes involved in signalling processes include 10 kinases, 5 PP2Cs and a membrane-bound ankyrin-like protein (LOC_Os02g50270) with a probable role in calcium-mediated signalling. From AraNet we find that the Arabidopsis ortholog of LOC_Os02g50270 is co-expressed with calmodulin genes, CAM3, 6 and 7. These Ca^2+^ sensors have been implicated in various developmental and environmental stimuli response [58]. The upstream promoter region of the ankyrin-like protein consists of ABRE elements, heat stress-responsive elements, and light responsive elements (e.g., G-box) indicating its possible role in stress. In our subnetwork we observe that its first neighbors are kinases involved in signalling and protein posttranslational modifications. The 10 kinases include cell-wall associated kinase 25, OsWAK25, involved in pathogenic resistance [59], chloroplast localized kinases (LOC_Os07g47270, LOC_Os01g71000 and LOC_Os03g06330) involved in redox regulation and phosphorylation [60], OsRPK1-receptor like kinase, a negative regulator of auxin transport [61] and receptor-like cytoplasmic kinase, OsRLCK-XV (LOC_Os06g07070) highly induced during drought and cold stress [62]. Topologically significant (high degree), the biologically relevant (co-clustered with ABA signalosome and connected to important kinases) along with ABA-responsive *cis*-elements in its promoter and potential role in calcium mediated signalling makes the ankyrin-like protein (LOC_Os02g50270) a likely novel candidate for drought stress. Cluster U1 also consists of two transcription factors (TFs), ABF-type OsbZIP23 and OsNAC6/SNAC2. The OsbZIP23 TF is associated with ABA signalling pathway and strongly induced by drought, high-salinity, polyethylene glycol (osmotic stress) and ABA-treatments. The interactions of OsbZIP23 with 7 genes of U10-cluster and 4 genes of U13-cluster in this subnetwork are in accordance with a genome-wide study using a ChIP assay by Zong et al [63]. Transgenic rice plants overexpressing OsNAC6/SNAC2 displayed increased sensitivity to ABA and tolerance to dehydration and high salinity stresses, suggesting its role as a transcriptional activator in both salinity and drought stresses [64–66]. Thus, we see that this subnetwork is able to capture apart from the ABRE regulon, role of NAC regulon in the transcriptional networks of abiotic stress response.

The rice SnRK2 kinases are shown to be over-expressed under abiotic stress with a probable role in phosphorylating ABF family of bZIP TFs for down-stream ABA signalling [67,68]. This association is well captured in our subnetwork exhibiting interactions of OsbZIP23 TF with SnRK2 kinases and PP2Cs (Cluster U1). The 5 ABF genes of Cluster U13 (red) are also seen to exhibit direct/indirect association with PP2Cs (Fig 3(B)) and their role in stress response is well documented. The OsbZIP66/TRAB1 gene encoding ABF-type TF (ABRE-binding bZIP factor) is reported to be up-regulated under dehydration and salt stress conditions [69]. The terminal or nearly terminal event of the primary ABA signal transduction pathway is phosphorylation of this gene [70,71]. The TFs OsbZIP45 and OsbZIP12 are also well characterized for their role in providing drought tolerance [72,73], while OsbZIP20/RISBZ3/RITA-1 is shown to be expressed in the late stages of seed development with a possibsle role in regulating seed-specific genes [74,75]. As both seed maturation and response to drought is co-regulated by ABA, the involvement of OsbZIP20 in drought tolerance can be further investigated. Similarly, OsbZIP86 being up-regulated and topologically significant in the co-expression network and its association with other stress-responsive bZIP TFs in the uDTN, suggest its possible role in stress response. These interactions suggest OsbZIP20 and OsbZIP86 transcription factors as probable novel candidates for drought stress.

The 10 kinase signalling genes (Cluster U1) are connected to 16 genes associated with primary metabolism and biosynthesis of secondary metabolites via Uridine 5'-monophosphate synthase, UMPS2 (LOC_Os01g72250). It is a precursor for pyrimidine nucleotides and is associated with the last step of de novo pathway of pyrimidine biosynthesis. Eleven of these 16 genes are involved in the biosynthesis of secondary metabolites associated with sugars and amino acids, and 5 genes belong to Pentose Phosphate Pathway (PPP). Role of PPP is to maintain redox potential to protect against oxidative stress by producing reductants such as NADPH [76]. To ensure these genes are possible targets of ABA-induced downstream signalling cascade, we carried out promoter analysis of these 17 genes (16 metabolic pathway genes+UMPS2). For this, 1 kb upstream region of these genes are analyzed using *cis*-acting regulatory elements database, PlantPAN (v2.0) [77]. The results indicate the presence of ABRE motifs like ‘ABRELATERD1’ in all the 17 genes upstream of the genes (S6 Table). The gene UMPS2 is up-regulated, has ABRE motifs in its promoter region and is topologically significant (‘hub’ gene) both in the co-expressed module and in uDTN. These results suggest the probable role of UMPS2 in relaying ABA-induced metabolic changes, hitherto unknown. To our knowledge there is only one proteomic study in which UMPS2 has been reported to be up-regulated in rice seedlings after ABA treatment followed by subsequent salt stress [78]. Thus, this analysis suggests UMPS2 as a potential drought-tolerant candidate gene in rice.

### Cluster U2: Molecular chaperons and heat stress transcription factors

Almost all stresses induce the production of a group of proteins called heat-shock proteins (Hsps) or stress-induced proteins as adverse environmental conditions disrupt protein folding and lead to an increase in reactive oxygen species (ROS) and oxidative stress within the cell [79,80]. Their transcription is controlled by regulatory proteins called heat stress transcription factors (Hsfs) by binding to the highly conserved heat shock elements (HSEs) in the promoter regions. The Hsps function as molecular chaperones, regulating cellular homeostasis and preventing protein misfolding and aggregation, thus promoting survival under stressful conditions. Cluster U2 in uDTN clearly captures these interactions. It contains 19 genes of which 7 are heat shock proteins (5 DnaJ/Hsp40 family and 2 DnaK/HSP70 family), 4 HsFs (OsHsfA9, OsHsfA2e, OsHsfB2c and OsHsfA2c), MYB TF (LOC_Os04g30890), CDK5RAP3 and another molecular chaperon, ERD1 protein (chloroplast precursor, LOC_Os02g32520) and involved in heat stress. The Hsp70s, assisted by co-chaperone DnaJ-proteins and nucleotide exchange factor constitute a chaperone machine that participates in protein folding, prevention of protein aggregation, translocation of proteins across membranes, targeting proteins towards degradation, and regulation of translation initiation [81,82].

Promoter analysis of the four HSFs revealed presence of ABRE elements suggesting their role in ABA-dependent signal transduction. It has been suggested that Hsps and Hsfs might be important elements in crosstalk of different stress signal transduction networks [83]. The first-degree neighbors of these TFs (extracted from STRING database) mapped to 13 genes in our co-expression network, with 10 genes being common neighbors to all the 4 TFs. Promoter analysis of the up-regulated neighbors (8) was carried out to identify possible regulatory elements in these transcriptionally active genes under drought. Majority of these genes are Hsps (LOC_Os09g29840, two Hsp70s, Hsp90, Hsp40, Hsp81-3, dnak—LOC_Os03g11910) and an RNA recognition motif family protein, LOC_Os03g15890. All the 8 genes have ABRE elements in their promoters, 5 of the genes have heat shock elements (HSEs) and 6 of them also have *cis*-elements which are responsive to methyl jasmonate (MeJa). These results indicate that the Hsps and HSFs are induced in an ABA-dependent manner and at the same time, they are also responsive to jasmonates indicating complex interplay between the two phytohormones. In a study by [84], it has been shown that OsHsfA2c (part of Turquoise module) binds to OsClpB-cyt/Hsp100 promoter (also part of Turquoise module) and possibly play the role of transcriptional activator in heat stress. Our analysis show that OsHsfA2c exhibits highest fold change, followed by OsHsfB2c, OsHsfA2e and OsHsfA9. Also, both ABRE and HSE elements are present in the promoter of OsClpB/Hsp100 gene, and is up-regulated in our network. These results suggest OsHsfA2c may play the role of transcriptional activator even in drought stress and the HSFs, OsHsfA2c, OsHsfB2c and OsHsfA2e are probable biomarkers for drought tolerance.

### Cluster U3, U4, U9 and U11: Components of amino acid metabolism, cell wall and TCA/Citric Acid Cycle and Mitochondrial ETC/ATP synthesis

**Fig 4:**
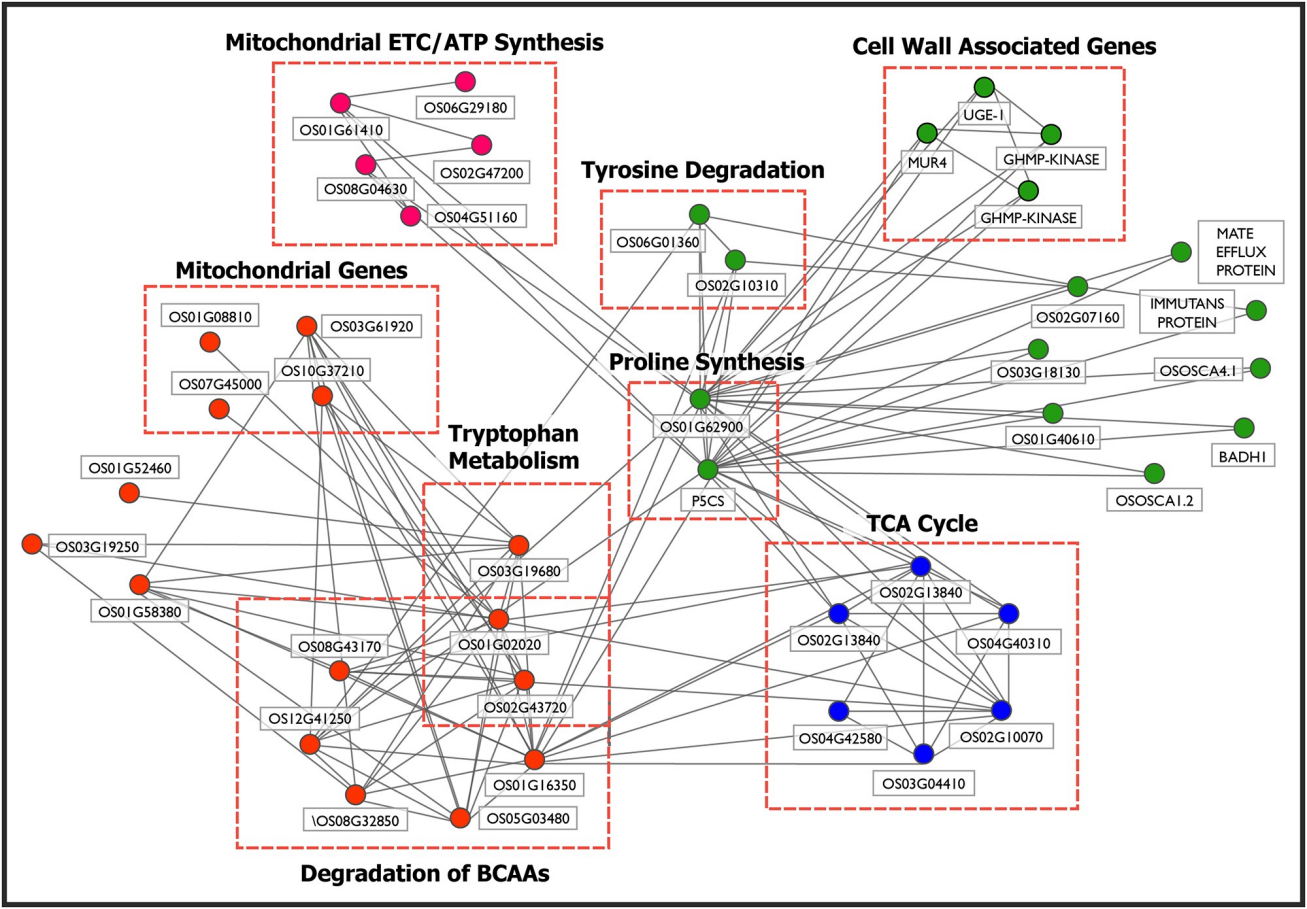
Subnetwork of Cluster U3 (‘green’), Cluster U4 (‘orange’), Cluster U9 (‘blue’) and Cluster U11 (‘pink’) obtained using MCL algorithm on up-regulated drought-tolerant network (uDTN) is shown. It depicts the crosstalk between the components of Amino Acid Metabolism (Cluster U3 and U4), Cell Wall metabolism (Cluster U3), TCA/Citric Acid Cycle (Cluster U9) and ATP synthesis (Cluster U4). These interactions indicate link between structural integrity of plant and cellular energy generating processes as a drought-adaptive mechanism.

Maintaining cellular elasticity and integrity by increased cellulose and hemicellulose synthesis is an important drought-tolerant trait [85,86]. We observe that these processes are captured by the subnetwork formed by Clusters U3 (Cell wall and amino acid metabolism), U4 (Amino acid degradation and mitochondrial ETC), U9 (TCA cycle) and U11 (Mitochondrial ETC/ATP synthesis), shown in Fig 4.

It is known from literature that Proline accumulation occurs under various stress conditions and imparts beneficial role to plants by maintaining osmotic balance, stabilizing membranes (thereby preventing electrolyte leakage) and in scavenging of free radical and ROS species [87,88]. Proline is also known to be a key determinant of many cell wall proteins that play an important role in cell wall signal transduction cascades, plant growth and differentiation. The interactions of amino acid kinases P5CS (LOC_Os05g38150) and LOC_Os01g62900, involved in proline biosynthesis with 4 genes involved in cell wall synthesis, OsUGE1 (UDP-glucose 4-epimerase 1), MUR4 (UDP-arabinose 4-epimerase), and GHMP kinases (LOC_Os02g04840 and LOC_Os03g61710) in Cluster U3, capture the role of proline as an important component of the cell wall matrix, providing tensile strength to the cell walls [89]. In Fig 4, we observe that the genes involved in proline biosynthesis in Cluster U3 is involved in crosstalks with clusters U4 (Amino acid degradation and mitochondrial ETC), U9 (TCA cycle) and U11 (Mitochondrial ETC/ATP synthesis).

Three genes of Cluster U3, glyoxalase family protein (LOC_Os02g07160), Fumarylacetoacetate (LOC_Os02g10310) and Homogentisate 1, 2-dioxygenase (LOC_Os06g01360) are involved in phenylalanine and tyrosine degradation. Fumarate (also a metabolite of citric acid cycle) and acetoacetate (3-ketobutyroate) are liberated in tyrosine catabolic process. Acetoacetate, on activation with succinyl-CoA, is converted into acetyl-CoA (LOC_Os01g02020 of Cluster U4), which in turn can be oxidized by citric acid cycle or used for fatty acid synthesis. This provides a crosstalk between genes of Clusters U3 (Cell wall and amino acid metabolism) and U4 (Amino acid degradation and mitochondrial ETC). Secondary metabolites such as lignins, flavonoids, isoflavonoids, etc. are usually derived from the catabolic pathway of tyrosine [90]. Accumulation of these phenolic compounds during drought is required for ROS scavenging. Moreover, increased lignin deposition, cell wall stiffness and reduced cell expansion is reported to inhibit plant growth [91–93]. Several stress-responsive genes such as hyperosmolality-gated calcium-permeable channels which act as osmosensors, OsOSCA1.2 (LOC_Os05g51630) and OsOSCA4.1 (LOC_Os03g04450) [94], MATE efflux family protein (LOC_Os03g42830) involved in transport [95], immutans protein (LOC_Os04g57320) involved in electron transport, and asparagine synthetase (LOC_Os03g18130) involved in nitrogen metabolism [96] are part of this subnetwork (Cluster U3) and connected to the amino acid kinases P5CS (LOC_Os05g38150) and LOC_Os01g62900, involved in proline biosynthesis suggesting transport and distribution of small metabolites.

The degradation of branched-chain amino acids (BCAAs) is elevated during stress to provide intermediates for TCA cycle and electron donors of the mitochondrial electron transport chain to generate cellular energy [97–99]. This process is captured by the six genes of Cluster U4, involved in the degradation of amino acids valine, leucine and isoleucine. The cluster also includes mitochondrial and peroxisomal genes, explaining the degradation of BCAAs occurring predominantly in these two cellular organelles [100]. Cluster U4 includes genes involved in electron carrier activity, *viz*., EFTA (LOC_Os03g61920), electron transfer flavoprotein subunit alpha-mitochondrial precursor and LOC_Os10g37210, FAD-dependent oxidoreductase domain containing protein (involved in electron transfer to mitochondrial respiratory chain). Membrane-bound cytochrome P450 genes (LOC_Os07g45000 and LOC_Os01g08810) with oxidoreductase activity are up-regulated and part of this cluster. Four out of 5 genes of Cluster U11 are also involved in mitochondrial electron transport chain (ETC)/ATP synthesis. This cluster shows an interaction between rice alternative oxidases OsAOX1b (LOC_Os04g51160), OsAOX1c (LOC_Os02g47200), NADH-ubiquinone oxidoreductases (LOC_Os08g04630, LOC_Os01g61410) and putative erythronate-4-phosphate dehydrogenase domain containing protein (LOC_Os06g29180). A number of roles have been associated with AOXs such as optimization of respiratory mechanisms, replenishment of TCA cycle intermediates [101], preventing excess generation of ROS [102] and shown to be up-regulated in response to biotic and abiotic stress conditions [103–106]. The genes of NADH-ubiquinone oxidoreductase complex in mitochondria are entry point to the respiratory chain from TCA cycle and are involved in ATP synthesis. The up-regulation of ATP synthesis is probably to meet energy demands of the plant to sustain metabolism under drought stress [107]. The putative homolog of LOC_Os06g29180 is an NAD+ -dependent formate dehydrogenase in Arabidopsis with a probable role in NADH synthesis [108]. Here too, we observe crosstalk between genes of Clusters U3 and U11 via amino acid kinases P5CS (LOC_Os05g38150) and LOC_Os01g62900 and NADH-ubiquinone oxidoreductases (LOC_Os08g04630, LOC_Os01g61410) linking proline catabolism inside mitochondria with a probable role in ROS production [109]

The TCA cycle takes place in mitochondria in a series of reactions to generate ATP. Five genes of Cluster U9 *viz*., citrate synthases (LOC_Os02g13840, LOC_Os02g10070), dehydrogenases (LOC_Os01g16900, LOC_Os04g40310) and aconitase (LOC_Os03g04410), are involved in the Citrate Cycle. Increase in the expression levels of these genes suggest an increase in ATP production as well as increase in reductants and substrates for non-essential amino acid synthesis like glutamine, proline, arginine, etc. [110–112]. Indeed, we do capture these interactions in the crosstalk between U4 (Amino acid degradation and mitochondrial ETC) and Cluster U9. The interactions between HMG-CoA (LOC_Os01g16350) and genes of the melavonate pathway (acetyl-CoA acetyltransferase (LOC_Os01g02020) and HMG-CoA synthase (LOC_Os08g43170)) in Cluster U4 with the genes of the TCA cycle (Cluster U9) provides a snapshot of the different metabolic fates of the acetyle-CoA, a central metabolite which can be incorporated into the TCA cycle for ATP generation in mitochondria, synthesis of amino acid carbon skeletons or metabolized in the cytosol to produce a plethora of phytochemicals required for growth, development and tolerance towards environmental stress [113,114].

### Smaller uDTN clusters

Apart from the major processes discussed above, several other smaller clusters identified in uDTN revealed various processes up-regulated in drought-stress, and are summarized in Table 4. For example, Cluster U5 is involved in RNA binding and harboring genes with RNA recognition motifs. RNA-processing via post-transcriptional modifications and alternative splicing are important components responsible for fine-tuning the regulatory mechanisms in response to environment stress [115–117]. Clusters U6 and U8 are involved in protein ubiquitination and degradation which are major posttranslational modifications, providing a dynamic and reversible control over processes such as degradation of misfolded proteins, trafficking, signal transduction and cell division [118,119]. Cluster U7 consists of genes associated with plant defense system. Other than stress-induced lipases and transporters, genes like CXE carboxylesterase (LOC_Os06g11090) and GID1L2-gibberellin receptors (LOC_Os11g13670 and LOC_Os05g33730) which are involved in biodegradation of xenobiotics (herbicides) are also present in this cluster. These genes are induced as a result of increased oxidative stress in the system [54] and may have a role in ROS scavenging in the plant. Starch synthesis and degradation is an important aspect regarding metabolic shifts during drought stress as seen in Cluster U13. It harbors alpha and beta-amylases involved in breakdown of starch and produce soluble sugars, OsDPE1 involved in transitory starch breakdown and AGPlar involved in starch synthesis. The increase in amylase activity in the shoot can be seen as an important drought tolerant trait, providing tensile strength and maintaining cellular membranes [120,121]. Thus we show that by using an integrated approach by combining co-expression and protein-protein interaction information we are able to capture some of the core biological processes in drought response. In the up-regulated PPIN, we are able to extract some of the key components of the ABA signalosome including the PP2Cs, the SnRK2 kinases and a number of ABF-type bZIP transcription factors. Along with the genes involved in signalling, we observe a crosstalk with the genes involved in metabolic pathways like Pentose Phosphate Pathway and biosynthesis of sugars and amino acids via the gene UMPS2, a novel candidate for drought. We also extracted a cluster comprising molecular chaperons and heat shock transcription factors as well as an MYB transcription factor co-clustered with these genes. The other major crosstalk is observed between processes, namely biosynthesis of proline which is an osmolyte and an important component of cell wall matrix, cell wall metabolism, degradation of branched chain amino acids to serve as intermediates for TCA cycle, enzymes of the TCA and finally, mitochondrial electron transport/ATP synthesis to generate cellular energy during stress. There is a re-partitioning of metabolites during drought stress as genes involved in starch biosynthesis as well as degradation are seen to be up-regulated. Finer regulatory mechanisms such as RNA-processing via post-transcriptional modifications and alternative splicing are captured in this network. Finally, we observe that protein ubiquitination and degradation is a prominent process in drought by which degradation of misfolded proteins due to stress, trafficking, signal transduction and cell division are dynamically controlled during stress.

### Analysis of dDTN

A similar analysis is carried out on the down-regulated drought tolerant network, dDTN, to identify the processes down-regulated in response to drought stress by the proposed network-based approach. Seventeen tightly-coupled gene clusters are identified using MCL algorithm in dDTN. For the clusters shown in Table 5 with a total of 449 genes, 465 edges have experimental evidences from model organisms (confidence score ≥ 0.4 in StringDB). Functional annotation for biological processes associated with these gene clusters using MapMan and RGAP is summarized in Table 5 and is discussed below in detail.

**Table 5:** Functional enrichment of 17 gene clusters identified in down-regulated drought tolerant network (dDTN) using MCL algorithm. (B: Blue, P: Purple, G: Green, M: Magenta, S: Salmon).

Cluster No.	Function	Total Genes	Cluster No.	Function	Total Genes
**D1**	Photosynthesis and associated process	262 (178B, 72R, 5S,3P, 4G)	**D8**	alpha-Linolenic acid metabolism	7 (4B, 3G)
**D2**	Cell Wall metabolism	45 (43M, 1B, 1G)	**D9**	Glutathione S-transferase	6 (4G, 2B)
**D3**	Signalling and post-translational modifications	37 (22G, 10B, 2R,1M, 1P, 1S)	**D10**	Serine Metabolism	6 (4B, 1P, 1M)
**D4**	Ribosome biogenesis	25 (12B, 8R, 4P, 1S)	**D11**	DNA-repair processes	5 (3B, 2P)
**D5**	tRNA biogenesis	22 (9B, 4G, 3R, 1S, 5P)	**D12**	Vitamin B6 metabolism	5 (2B, 2G, 1R)
**D6**	Development and Flowering	11 (4B, 7G)	**D13**	Cell vesicle transport	5 (2G, 2M, 1B)
**D7**	Signalling and Transport	8 (5B, 1M, 1S,1G)	**D14**	Biotic Stress	5 (3G, 1B, 1P)

### Cluster D1: Photosynthesis and associated processes

Photosynthesis, a part of the primary metabolic process in plant, plays a central role in drought response. With decrease in carbon uptake due to stomatal closure, major metabolic shifts take place to re-partition the photo-assimilates between root and shoot, which lead to the cessation of shoot growth and maintenance of root growth under water deficit conditions [122]. Around ~23% of Cluster D1 genes are directly involved in photosynthesis, *viz*., genes associated with photosynthetic light reaction such as ATP synthases and NADPH dehydrogenase subunits, chloroplast precursors and chlorophyll A-B binding proteins. Closely associated processes with photosynthesis such as carbon fixation, tetrapyrrole synthesis and carbohydrate metabolism are also components of this cluster. Closely clustered with the photosynthetic genes, around 30% genes are involved in protein synthesis, folding, targeting, posttranslational modifications and degradation. On closer look, a large majority of them are ribosomal proteins, many of which are located in chloroplast (21 genes). Genes associated with RNA processing (helicases), regulation and binding (52 genes) are also seen to be clustered together with photosynthetic processes. The crosstalk between these processes suggests down-regulation of photosynthesis and transcription and translation of plastidial genes.

Twenty one genes in this cluster have no functional annotation. These genes are queried in AraNet and the top functional predictions for each gene are listed in S6 Table. Of these, ~20 genes have predicted functions associated with photosynthetic machinery, pathogen response or both. Promoter analysis of these 21 uncharacterized genes using PlantPan database revealed presence of cis-elements like ‘GT1CONSENSUS’ and ‘CGACGOSAMY3’ indicating the presence of light-responsive elements like G-box in all the genes. These elements are known to regulate light-specific gene expressions and response to stimuli [123]. Other motifs like “SITEIOSPCNA” which is shown to be involved in gibberellin-responsive pathway in regulating photosynthesis [124] are also present. The above analyses indicate that the 21 uncharacterized genes of this cluster that are topologically significant and tightly clustered with photosynthetic genes, are likely to be regulated by light, respond to pathogen invasion/wounding and may be influenced by hormones such as gibberellin. We presume these genes to be important candidates for transgenic studies to elucidate their role in photosynthesis and drought-response.

### Cluster D2, cell wall metabolism

Plant cell walls are of critical importance and provide cell shape and mechanical support to withstand the turgor pressure. However, during water deficit, aerial growth is limited due to reduced cell division in the meristematic zones [125] followed by reduced ability of cells to expand the polysaccharide network. Cluster D2 genes are found to be associated with cell wall metabolism, e.g., cellulose synthases OsCESA6, 7 and 8 and cellulose synthase-like genes OsCLSA1 and OsCLF6, and OsBC1L4/OsCOBRA involved in cellulose biosynthesis. While OsCESA6, 8 and OsBC1L4/OsCOBRA are reported to be involved in primary cell wall biosynthesis [126], OsCESA7 is a secondary cell wall-specific cellulose synthase [127]. The gene OsCLF6 is shown to mediate mixed-linkage glucans (MLG) synthesis in rice [128]. MLG is a cell wall polysaccharide, known to be involved in the regulation of cell wall expansion in young tissues [129,130]. This cluster also includes 3 Fasciclin-like arabinogalactan proteins (OsFLA6, 16 and 24) that are involved in cell wall adhesion, signalling, biosynthesis and remodeling and in the development of new shoot and root meristems [10]. Among the 5 uncharacterized genes, we observe that Arabidopsis orthologs of LOC_Os06g11990 and LOC_Os05g32500 are involved in cellulose synthesis while that of LOC_Os07g43990 has a role in transferring glycosyl groups (S6 Table). Since cell walls are also the first line of defense against pathogens, some of the genes, e.g., Harpin-induced protein 1 (LOC_Os02g33550) and CHIT1 (LOC_Os09g32080- Chitinase family protein) are down-regulated and are part of this cluster. Some of the kinases (LOC_Os02g43870, LOC_Os03g12250 and LOC_Os04g55620) have LRR domains and are important for protein-protein interactions, signalling and response to biotic stress. Thus, a low turgor pressure during drought stress leads to reduction of growth by the inhibition of cell wall elongation. The down-regulation of Cluster D2 genes clearly reveals the processes associated with cell wall biosynthesis that are down-regulated in leaves.

### Cluster D3, D7 and D14: Down-regulation of biotic stress associated genes

The natural phenolic compound, salicylic acid (SA) is one of the main hormones involved in endogenous signalling in plant pathogen response and disease resistance [131]. In many studies drought tolerance and disease resistance pathways are reported to be antagonistic and the role of ABA in suppressing SA-signalling pathway [132–134]. A transcriptional activator NPR1 is an important regulator of SA-mediated defense signalling pathway and responsible for activation of a large number of downstream pathogen-responsive genes [135,136]. It is shown that ABA suppresses transcriptional up-regulation of WRKY45 and OsNPR1, the two important components of SA-signalling pathway [137]. Also, OsNPR1 has been implicated with reduced drought tolerance in rice [138]. Both WRKY45 and OsNPR1 (part of Cluster D3) are co-expressed in Green module and down-regulated in agreement with earlier studies, thus capturing the crosstalk between ABA and SA signalling pathways.

Majority of Cluster D3 genes (~ 23) are kinases including receptor-like kinases involved in signalling and post-translational modifications. Many of these genes are shown to be involved in plant immunity against pathogens and are potential pattern recognition receptors (PRRs), e.g., leucine-rich repeats (LRR) and ankyrin (ANK) repeats [139–142]. This module consists of three LRR repeat genes, Strubbelig-receptor FAMILY8 precursor (LOC_Os06g42800), a putative transmembrane protein kinase 1, a putative protein kinase (LOC_Os01g41870) and Brassinosteroid insensitive 1-associated receptor kinase 1 precursor (BAK1, LOC_Os11g39370). Previous studies indicate that these kinases influence cellular morphogenesis, orientation, cellular proliferation, size and shape [143–145]. Genes with ankyrin repeats include OsNPR1 (LOC_Os01g09800) a master regulator characterized for resistance to plant pathogens [146–148] and LOC_Os03g63480, are both involved in biotic stress and down-regulated in the network.

Three disease resistance proteins *viz*., RGA3, RPM1 and LOC_Os12g25170 with NB-ARC domains containing LRR repeats and having ATPase and nucleotide binding activities are part of Cluster D14. These proteins are involved in pathogen resistance and help in discriminating between self and non-self (R-protein mediated response) [149]. Cluster D7 consists of 2 genes (OsGF14b and OsGF14e) that belong to 14-3-3 gene family. These signalling proteins are reported to be involved in biotic stress and probably associated with JA and SA pathways [150,151]. They interact with Beta-2- and Beta-5-tubulin proteins which play critical role in cell division, elongation and growth. Binding of 14-3-3-signalling proteins with those involved in cellular organization (tubulins) hint at finer regulations of cell cycle events [152]. Thus, the analysis of the subnetwork of Cluster D3, D7 and D14 indicate suppression of the defense system of plants under drought stress, that is, the vulnerability of the plant to pathogen infection under drought condition.

### Cluster D4 and D5: Biogenesis of rRNA and tRNA genes

A number of clusters are associated with synthesis, post-translational modifications and processing of ribosomal proteins. Cluster D4 includes genes involved in pre-rRNA processing in nucleolus and cytoplasm (nucleolar protein 5A-LOC_Os03g22880, LOC_Os04g49580 and Gar2-LOC_Os08g09350), ribosomal proteins (LOC_Os08g41300, LOC_Os06g03790, LOC_Os06g02510 and LOC_Os03g41612), DEAD/DEAH box helicases and WD domain containing proteins, t-RNA synthetases (LOC_Os05g08990 and LOC_Os04g02730) and other RNA-binding genes. The rRNA processing and ribosome biogenesis is tightly coupled with growth and cell proliferation. Down-regulation of these pathways hint at conservation of energy by the plant [153,154]. Closely interacting with Cluster D4, is Cluster D5 with genes involved in amino-acyl tRNA biosynthesis (8 genes), genes involved in protein-processing like ubiquitin conjugating enzymes (LOC_Os01g46926, LOC_Os04g58800, LOC_Os06g44080 and LOC_Os03g03130), further indicating a decrease of protein turnover, cell proliferation and growth.

### Cluster D6: Development and flowering

Cluster D6 consists of zinc finger domain containing proteins-LOC_Os04g46020, OsTIFY1b, OsBBX25, zinc finger protein-LOC_Os08g15050, MADs box genes (OsMADS50 and OsMADS55) and response-regulator genes (OsRR10 type-A and OsRR2 type-A). The TIFY family genes are involved in development and have been shown to be stress-responsive [155]. The MADs box and BBX genes are also involved in flowering and development [156–158]. Interestingly, rice response regulator (OsRR) genes are part of the cytokinin-dependent signal-transduction pathways which also affect development and morphological changes in the plant [159,160]. The down-regulation of genes in this cluster suggests a decrease in growth, development and delay in flowering of the plant under drought stress.

### Cluster D8: α-Linolenic acid metabolism

The phytohormone Jasmonic Acid (JA) regulates a wide array of biological processes in plants including growth, development, secondary metabolism, pathogen defense, wounding as well as tolerance to abiotic stress [161]. Two genes of GH3 family, *viz*. OsJAR1 and OsJAR2 control the homeostasis of JA by forming bioactive JA-amino acid conjugates. They have been reported to be involved in wound and pathogen-induced JA-signalling [162]. Also, OsOPR7, LOX7 and OsAOS1 genes that are involved in JA synthesis [163,164] are down-regulated and part of this cluster. The wound inducible and leaf-specific gene, OsHPL3 of this cluster is reported to modulate the amount of JA by consuming common substrates [165]. As majority of the genes in JA biosynthesis are down-regulated, it can be inferred that during drought stress, there is an increased susceptibility to wounding and pathogen attack.

### Other smaller dDTN clusters

Apart from some of the major clusters discussed above, several smaller clusters are detected (Table 5). For example, Cluster D9 consists of six glutathione s-transferases (GSTs). The GST family of genes are widely involved in cellular detoxification and can act on substrates such as endobiotic and xenobiotic compounds [166]. However, studies have indicated that not all the GST genes express the same way across different stress, tissue and developmental conditions [167]. A similar behavior is observed in the present case with GSTs in D9 are down-regulated, but 12 other GSTs are up-regulated in the overall co-expression network, and are not part of uDTN. Cluster D10 consists of genes associated with serine metabolism and are down-regulated. Among these, two genes are phosphoserine phosphatase which catalyzes the last step in the plastidial phosphorylated pathway of serine biosynthesis. The other genes include serine hydroxymethyltransferase (SHMT) gene and three aminotransferases with a probable role in glycine cleavage system. The glycine cleavage system is required for photorespiration in C3 plants and closely interacts with serine SHMT for the methylation of glycine to serine. These amino acids have been associated with photorespiration and precursors for other metabolites involved in stress protection [168]. It has been shown that DNA repair mechanisms are affected by drought stress. This is captured by Cluster D11 genes involved in maintaining genome stability and repair mechanisms. With increase in ROS due to stress, plants undergo ABA-mediated growth retardation with increase in DNA damage, inhibition of DNA replication and cell division [169–171]. Genes involved in vitamin B6 metabolism are captured in Cluster D12. Lower levels of vitamin B6 levels have been attributed to impairment of flowering and reduced plant growth in Arabidopsis mutants [172,173]. The Cluster D13 consists of genes 3 major sperm proteins (MSPs) which have not been studied in the context of stress response. These MSPs have a probable role in cell vesicle transport and mediate protein-protein interactions within or between cells [167]

## Discussion

The fascinating complexity of biological processes in plants is reflected in the intricate interactions in co-expression networks. With the availability of a large number of transcriptomic datasets in public repositories, co-expression network analysis allows us to integrate this information and perform large-scale multi-genic studies. On one hand, we need this complexity to infer functional annotations of genes from tightly connected neighborhood (‘guilt-by-association’), while on the other hand, we need to dissect the networks to remove noisy connections and identify critical biological processes. Various approaches have been proposed, e.g., construction of condition-dependent co-expression networks with context-specific interactions [174,175], split co-expression networks into functional modules which may represent tightly coordinated biological processes [176,177], use of functional and comparative genomics [178,179], etc.

Here we propose a computational approach using network parameters that can help in identifying important drought specific processes. However, a typical meta-analytic study based on publicly available microarray datasets is likely to have heterogeneity in the data arising from various sources such as microarray platforms, tissues, developmental stages, experimental conditions, etc. Hence, for the analysis DEGs are restricted to only the 10 drought-responsive modules, which are further filtered based on topological measures, *viz*., intramodular connectivity (K_IM_) and eigengene-based centrality (K_ME_) to identify biologically significant genes across the genotypes. We observe that greater than 50% of these topologically significant DEGs are well represented in majority of the drought-tolerant genotypes considered (S10 Table), clearly suggesting their relevance. Using this filtered set of DEGs, two PPI networks are constructed by extracting known protein-protein interactions (from STRING, AraNet and RiceNet) within the set of up-regulated and down-regulated genes. Tightly-coupled gene clusters identified are identified using Markov clustering: uDTN (466 nodes, 1015 edges) and dDTN (665 nodes, 8719 edges). It is confirmed that these gene clusters are well-represented across all the 9 data subsets considered (S12 and S13 Tables).

**Fig 5:**
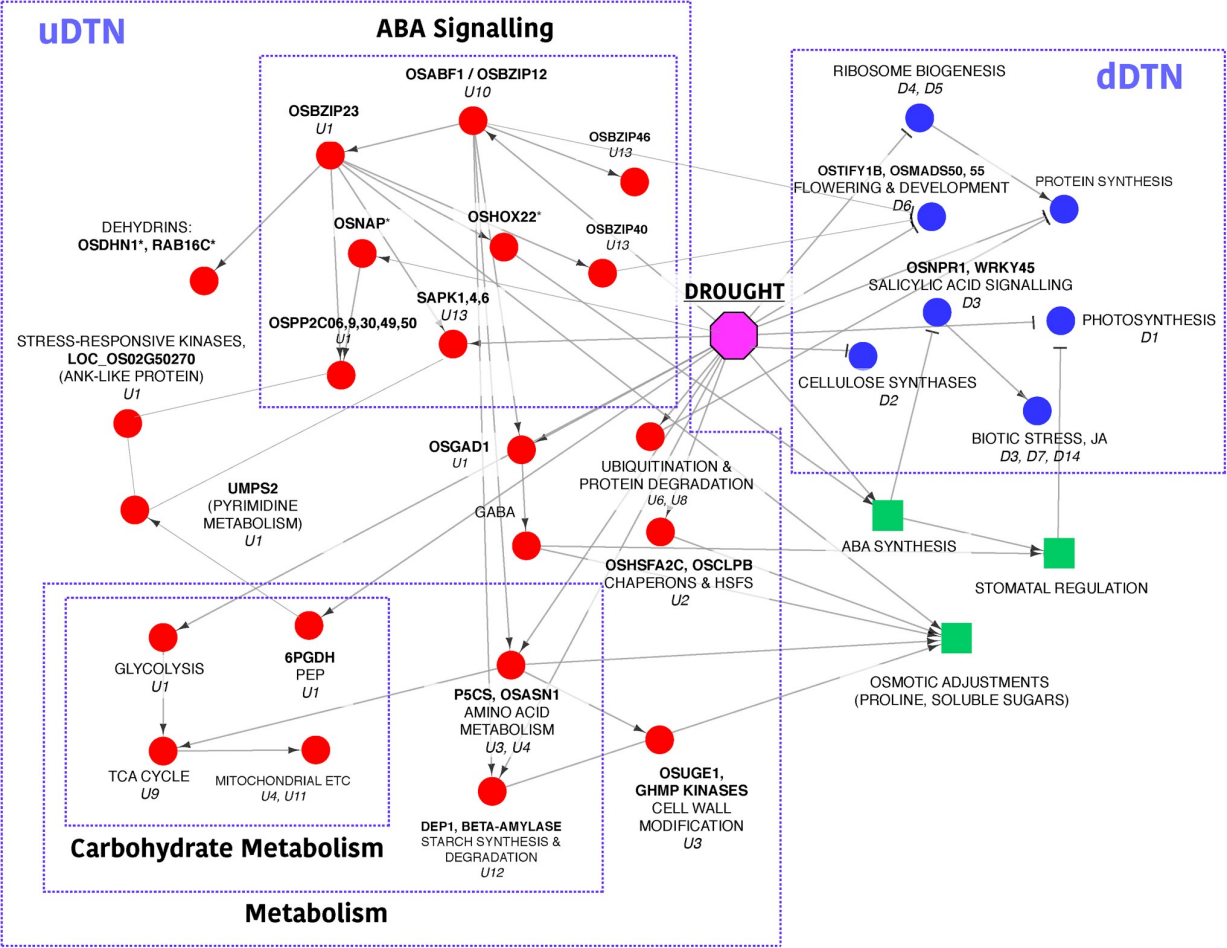
A representative model for drought-responsive mechanisms in drought-tolerant genotypes. Red nodes indicate key genes and clusters up-regulated and part of uDTN. Blue nodes indicate key genes and processes down-regulated and part of dDTN. Green nodes indicate key processes affected by uDTN genes. Nodes with ‘*’ indicate uDTN seed genes.

Plant’s response to drought stress involves complex interactions across several layers leading to molecular, biochemical, physiological and morphological changes in the plant. By our proposed computational approach, we identified various up- and down-regulated genes/gene clusters that are likely to play an important role in drought responsive processes. Functional characterization of these gene clusters, aided by interaction databases (such as STRING DB, RiceNet and AraNet) and additional information curated from literature support the proposed systems-level model for drought stress response in Fig 5. We observe that the key players in providing drought tolerance are bZIP TFs (mainly bZIP12 (U10), bZIP23 (U1), and bZIP46 (U13), ABA signalling machinery and interaction of its signalling components with metabolic pathways. From previous studies it is well known that ABA signalling pathway is triggered in response to drought stress [180–183]. The interaction between various bZIPs is seen to connect the three clusters U1, U10 and U13 (that are up-regulated across all the three stages of plant (S12 Table), and capture the ABA signalosome complex (Fig 1).

In over-expression experiments, OsbZIP23 TF (U1) has been shown to regulate genes with roles in drought tolerance, such as Dehydrins (OsDhn1, RAB16C), OsbZIP40 (U13) and Oshox22 (involved in ABA biosynthesis [184]) and an increased concentration of osmolytes such as proline and soluble sugars observed [185]. These genes are all part of our predicted uDTN and provide drought-tolerant traits. The interactions between these genes represent the mechanisms providing drought tolerance are depicted in Fig 5.

The OsbZIP12 (Cluster U10) regulates a large number of genes involved in ABA signalling and in metabolic pathways [186] and provides link between these two important mechanisms in drought response (depicted in Fig 5). It has been reported that its major targets are OsbZIP23 (U1), OsbZIP46 (U13) and OsGAD1 (associated with regulation of stomatal closure during drought stress [187], polyamine and proline accumulations [188]) and various PP2C genes (OsPP2C09, OsPP2C30 and OsPP2C49), in a study on transgenic rice lines using RNA-seq and Chip-seq assays by Zang *et al*. [42], further providing evidence to the interactions captured by our model. Metabolic pathway genes (4-alpha-glucanotransferase (DEP1) and beta-amylase (OsSTA246)) involved in degradation of starch into soluble sugars (Cluster U12) are known to play an important role in osmotic adjustments [189] and are also regulated by OsbZIP12 [42].

The bZIP TFs, OsbZIP12 and OsbZIP40 (a probable target of OsbZIP23), have been indicated as negative inhibitors of floral initiation [190]. This interaction is represented in Fig 5, linking the bZIP TFs to the down-regulated Cluster D6, capturing flowering and developmental processes and provides a probable drought escape mechanism [191]. Stress avoidance mechanisms involve dynamic changes in protein and metabolite abundances. Although a direct correlation with transcriptomic changes is a challenge owing to the intervening post-translational and other regulatory mechanisms, snapshots of these events are indeed captured by our meta-analysis. For example, Arginine, Proline and Asparigine metabolisms are up-regulated (U3, U4) resulting in an increase in free amino acid level. This has been reported in many drought tolerant lines from various plant species [192,193]. A similar pattern of protein degradation and ubiquitination is observed (U6, U8) and protein synthesis likely to be affected due to down-regulation of ribosome biogenesis (D4, D5).

Carbohydrate metabolism (Glycolysis (U1), TCA cycle (U9) and Pentose Phosphate Pathway (U1)) plays a key role in drought response. These pathways aid in energy production under adverse conditions, sugar accumulation, providing carbon skeletons for primary and secondary metabolites, and have been reported to be drought-tolerant mechanisms [194,195]. From Fig 3, their association with ABA signalling can be noticed. The oxidative branch of PEP links carbohydrate and fatty acid as well as purine and pyrimidine nucleotide metabolisms. The PEP pathway has been categorized as a “metabolic sensor” in response to oxidative stress and at the same time provides precursors for *de novo* biosynthesis of pyrimidines via PRPP [196], thereby linking UPMS2 to ABA signalling. The presence of ABRE *cis* elements in UMPS2 and its 16 neighbours of the metabolic components (Fig 3) further confirm this fact. As such, it forms a ‘hub’ and can be explored further as a novel candidate for drought tolerance. Other protective mechanisms are provided by heat shock proteins and transcription factors (HSPs and HSFs). Presence of ABRE elements and *cis*-elements associated with methyl-jasmonate (MeJa) (U2) in these up-regulated proteins indicate a protective role in cellular homeostasis, ROS scavenging as well as in osmotic adjustments.

Photosynthesis is a key process of the primary metabolism. A number of associated processes such as stomatal closure with reduced CO_2_ diffusion, induction of senescence by ABA, and rolling of leaves leading to reduced surface area contribute towards reduced photosynthetic rates [197]. In the present study, genes associated with photosynthetic light reaction such as ATP synthases and NADPH dehydrogenase subunits, chloroplast precursors and chlorophyll A-B binding proteins, etc. were found to be down-regulated as a result of drought stress. Antagonistic interactions between ABA and NPR1 and WRKY-45 dependent SA signalling pathway (D3) lead to the down-regulation of several disease resistance genes (D7, D14) and potential PRRs (kinases) with LRR and ANK repeats (D3). Additionally, genes involved in JA homeostasis are seen to be down-regulated, thereby making the plant susceptible to wounding and pathogen attack. Majority of the signalling and transport related genes (D7) down-regulated across all the 9 data subsets indicate susceptibility to biotic stress as well as growth arrests under drought stress. Finally, as expected, genes involved in cellulose synthesis and in primary and secondary metabolism (D2) are down-regulated under drought stress. At the same time, a compensatory mechanism between proline biosynthetic genes (P5CS) and cell wall-related genes (U3), and increase in secondary metabolites are observed in uDTN.

Thus, we see that the proposed integrated network-based approach is helpful in identifying important drought responsive processes that are well-represented in majority of the data subsets considered. Moreover, *in silico* functional annotations of uncharacterized genes, e.g., membrane-bound ankyrin-like protein (LOC_Os02g50270) associated with ABA signalling in uDTN, uncharacterized genes associated with known photosynthetic genes and light-responsive promoters, as well as those associated with cellulose synthesis are identified as important drought-responsive biomarkers. Further analysis of these genes using transgenic studies may help in elucidating their role in drought response.

## Conclusion

In this study, a comprehensive analysis of the complex drought-responsive processes using an integrated, systems-level approach across seven drought tolerant rice genotypes is presented. Preliminary analysis of the co-expression network revealed that transcriptional regulatory processes, post-translational modifications followed by photosynthesis are at the forefront of drought response. With the integration of protein-protein interactions, a detailed view of the system is possible and critical processes which otherwise lay hidden are brought forth. The phytohormone abscisic acid is seen to play a central role in regulating the energy metabolism of cells. Protective mechanisms like the transcriptional activation of molecular chaperons, amino acid metabolism and cellular respiratory processes are enhanced. The down-regulation of photosynthetic machinery and cellulose-associated genes explains a cessation of aerial growth during drought stress. The antagonistic relationship observed between ABA and SA-dependent pathogen response pathways indicate increased susceptibility of plants to certain pathogens during drought.

The systems-based approach discussed above, apart from capturing important stress-responsive processes, also help in *insilico* functional annotation of uncharacterized genes. Based on neighborhood analysis in the co-expression and protein-protein interaction networks, important biomarkers with significant topological properties and connectivity to other stress-responsive genes have been identified. A systematic dataset-specific analysis revealed that certain processes are well-conserved across all genotypes/stages, while some exhibit genotype-specific variations. Drought resistance/tolerance is indeed a complex process with dependencies on many variables, *viz*., soil, weather conditions, duration of stress, tissue-specific response, etc. Better designed experiments with larger sample sizes and taking these variables into consideration will further enhance the efforts in reliable detection of drought-tolerant biomarkers.

## Supporting information

S1 Table Dataset details. (XLSX)

S2 Table Co-expression network with genes and module labels. (XLSX)

S3 Table Module preservation statistics. (XLSX)

S4 Table Up-regulated PPIN (uDTN). (XLSX)

S5 Table Down-regulated PPIN (dDTN). (XLSX)

S6 Table Analysis of cis-elements for some of the clusters. (XLSX)

S7 Table Stage-specific DEGs across the datasets. (XLSX)

S8 Table Distribution of DEGs in the 6 microarray studies across 9 data subsets. (DOCX)

S9 Table Distribution of DEGs from up and down-regulated drought-responsive modules in the 6 microarray studies across 9 data subsets. (DOCX)

S10 Table Distribution of the DEGs (after screening for important genes) used for the construction of uDTN and dDTN shown across 9 data subsets. (DOCX)

S11 Table Distribution of uDTN and dDTN DEGs shown across 9 data subsets (after extracting PPIs). (DOCX)

S12 Table Distribution of DEGs in uDTN clusters. (DOCX)

S13 Table Distribution of DEGs in dDTN clusters. (DOCX)
