# Supplementary material for: Meta-analysis of drought-tolerant genotypes in Oryza sativa: A network-based approach
Source: PLoS One. 2019 May 6;14(5):e0216068. doi: 10.1371/journal.pone.0216068 (PMC6502313; doi:10.1371/journal.pone.0216068)
Supplement: S8 Table — (DOCX) [file pone.0216068.s008.docx]

**Table S8: Distribution of DEGs in the 6 microarray studies across 9 data subsets**

| **DEGs** | **Vegetative-seedlings** | | | **Vegetative-Leaves** | | | **Reproductive phase** | | | **No. of Data subsets ≥ 50% DEGs** |
| --- | --- | --- | --- | --- | --- | --- | --- | --- | --- | --- |
|  | **GSE41647** | **E-MEXP-2401** | **GSE21651**  **(only leaf)** | **GSE26280 (Tillering)** | **GSE24048 (Azucena)** | **GSE24048 (Bala)** | **GSE26280 (PE)** | **GSE25176 (Flag leaf)** | **GSE26280 (booting)** |  |
| **Up:**  **3082** | **2178**  **(70.7)** | **1149**  **(37.3)** | **1611**  **(52.3)** | **1923**  **(62.4)** | **1744**  **(56.6)** | **1594**  **(51.7)** | **2326**  **(75.5)** | **1324**  **(43)** | **2121**  **(68.8)** | **7** |
| **Down:**  **3372** | **2168**  **(64.3)** | **2366**  **(70)** | **1634**  **(48.4)** | **1454**  **(43)** | **1658**  **(49)** | **1354**  **(40)** | **2597**  **(77)** | **1652**  **(49)** | **1655**  **(49.1)** | **3** |
