# Supplementary material for: Meta-analysis of drought-tolerant genotypes in Oryza sativa: A network-based approach
Source: PLoS One. 2019 May 6;14(5):e0216068. doi: 10.1371/journal.pone.0216068 (PMC6502313; doi:10.1371/journal.pone.0216068)
Supplement: S9 Table — (DOCX) [file pone.0216068.s009.docx]

**Table S9: Distribution of DEGs from up and down-regulated drought-responsive modules in the 6 microarray studies across 9 data subsets. Almost all the DEGs are represented in these 10 modules indicating their significance in drought response.**

**TQ: Turquoise, Y: Yellow, Br: Brown, Ta: Tan, Bl: Blue, G: Green, M: Magenta, P: Purple, R:Red, S: Salmon**

| **DEGs** | **Total No. of DEGs** | **Vegetative Phase - Seedlings** | | | **Vegetative Phase – Leaves** | | | **Reproductive Phase** | | | **No. of Data subsets**  **≥ 50% DEGs** |
| --- | --- | --- | --- | --- | --- | --- | --- | --- | --- | --- | --- |
|  |  | **GSE41647** | **E-MEXP-2401** | **GSE21651**  **(only leaf)** | **GSE26280 (Tillering)** | **GSE24048 (Azucena)** | **GSE24048 (Bala)** | **GSE26280**  **(PE)** | **GSE25176 (Flag leaf)** | **GSE26280**  **(Booting)** |  |
| **Up**  **(in TQ+Y+**  **Br+Ta)** | 3012 | 2163  (72) | 1127  (52) | 1581  (52.5) | 1869  (62.1) | 1720  (57.1) | 1547  (51.4) | 2279  (75.6) | 1313  (44) | 2073  (69) | 8 |
| **Down**  **(in Bl+G+**  **M+P+R+S)** | 3235 | 2064  (64) | 2262  (70) | 1581  (47.3) | 1431  (44.2) | 1612  (50) | 1311  (40.5) | 2514  (77.7) | 1617  (50) | 1631  (50.4) | 6 |
