# Supplementary material for: Meta-analysis of drought-tolerant genotypes in Oryza sativa: A network-based approach
Source: PLoS One. 2019 May 6;14(5):e0216068. doi: 10.1371/journal.pone.0216068 (PMC6502313; doi:10.1371/journal.pone.0216068)
Supplement: S10 Table — (DOCX) [file pone.0216068.s010.docx]

**Table S10: Distribution of the DEGs (after screening for important genes) used for the construction of uDTN and dDTN shown across 9 data subsets. It may be noted that each of the 9 data subsets have ≥ 50% representation of the up- and down-regulated DEGs.**

| **DEGs** | **No. of DEGs** | **Vegetative Phase - Seedlings** | | | **Vegetative Phase – Leaves** | | | **Reproductive Phase** | | | **No. of Data subsets**  **≥ 50% DEGs** |
| --- | --- | --- | --- | --- | --- | --- | --- | --- | --- | --- | --- |
|  |  | **GSE41647**  **(DD)** | **E-MEXP-2401 (N22)** | **GSE21651**  **(Vandana,only leaf)** | **GSE26280 (DK151-Tillering)** | **GSE24048 (Azucena)** | **GSE24048 (Bala)** | **GSE26280**  **(DK151-PE)** | **GSE25176 (IRAT109Flag leaf)** | **GSE26280**  **(DK151-Booting)** |  |
| **Up**  **(%)** | 754 | 683  (91.6) | 391  (52) | 574  (76) | 599  (79.4) | 535  (71) | 468  (62.1) | 677  (90) | 475  (63) | 668  (89) | 9 |
| **Down**  **(%)** | 893 | 687  (77) | 709  (79.3) | 610  (68.3) | 484  (54.2) | 557  (62.4) | 441  (49.3) | 830  (93) | 625  (70) | 600  (67.2) | 8 |
