# Supplementary material for: Meta-analysis of drought-tolerant genotypes in Oryza sativa: A network-based approach
Source: PLoS One. 2019 May 6;14(5):e0216068. doi: 10.1371/journal.pone.0216068 (PMC6502313; doi:10.1371/journal.pone.0216068)
Supplement: S11 Table — (DOCX) [file pone.0216068.s011.docx]

**Table S11: Distribution of uDTN and dDTN DEGs shown across 9 data subsets (after extracting PPIs). It may be noted that each of the 9 data subsets have ≥ 50% representation in both uDTN and dDTN.**

| **PPIN** | **No. of DEGs** | **Vegetative Phase - Seedlings** | | | **Vegetative Phase – Leaves** | | | **Reproductive Phase** | | | **No. of Data subsets**  **≥ 50% DEGs** |
| --- | --- | --- | --- | --- | --- | --- | --- | --- | --- | --- | --- |
|  |  | **GSE41647(DD)** | **E-MEXP-2401**  **(N22)** | **GSE21651**  **(Vandana.only leaf)** | **GSE26280 (DK151-Tillering)** | **GSE24048 (Azucena)** | **GSE24048 (Bala)** | **GSE26280**  **(DK151-PE)** | **GSE25176 (IRAT109Flag leaf)** | **GSE26280**  **(DK151-Booting)** |  |
| **uDTN**  **(%)** | 466 | 427  (91.6) | 255  (59.7) | 354  (76.0) | 373  (80) | 327  (70.2) | 294  (63.1) | 419  (89.9) | 287  (61.6) | 416  (89.3) | 9 |
| **dDTN**  **(%)** | 665 | 542  (81.5) | 501  (75.3) | 478  (71.9) | 365  (57.6) | 381  (54.9) | 281  (42.2) | 630  (94.7) | 447  (67.2) | 454  (68.3) | 8 |
