# Supplementary material for: Meta-analysis of drought-tolerant genotypes in Oryza sativa: A network-based approach
Source: PLoS One. 2019 May 6;14(5):e0216068. doi: 10.1371/journal.pone.0216068 (PMC6502313; doi:10.1371/journal.pone.0216068)
Supplement: S12 Table — (DOCX) [file pone.0216068.s012.docx]

**Table S12 Distribution of DEGs in gene clusters shown. All uDTN clusters have ≥ 50% representation from 6 or more data subsets (except 1).**

| **uDTN**  **Clusters** | **No. of DEGs** | **Vegetative-Seedlings** | | | **Vegetative-Leaves** | | | **Reproductive phase** | | | **Data subsets**  **≥ 50% DEGs** |
| --- | --- | --- | --- | --- | --- | --- | --- | --- | --- | --- | --- |
|  |  | **GSE41647**  **(DD)** | **E-MEXP-2401**  **(N22)** | **GSE21651**  **(Vandanaonly leaf)** | **GSE26280 (DK151-Tillering)** | **GSE24048 (Azucena)** | **GSE24048 (Bala)** | **GSE26280 (DK151-PE)** | **GSE25176**  **(IRAT109-Flag leaf)** | **GSE26280 (DK151-Booting)** |  |
| ABA-signaling & secondary metabolism (U1) | 35 | 91.4 | 51.4 | 85.7 | 77.1 | 74.3 | 68.6 | 97.1 | 62.9 | 91.4 | 9 |
| Molecular chaperons & heat stress TFs  (U2) | 19 | 89.5 | 57.9 | 63.2 | 73.7 | 57.9 | 52.6 | 89.5 | 42.1 | 84.2 | 8 |
| Cell wall & amino acid metabolism  (U3) | 16 | 100.0 | 75.0 | 81.3 | 87.5 | 81.3 | 62.5 | 100.0 | 75.0 | 100.0 | 9 |
| Amino acid degradation & mitochondrial ETC  (U4) | 15 | 93.3 | 40.0 | 80.0 | 93.3 | 73.3 | 46.7 | 93.3 | 53.3 | 86.7 | 7 |
| RNA binding & processing  (U5) | 13 | 46.2 | 23.1 | 38.5 | 92.3 | 92.3 | 61.5 | 46.2 | 76.9 | 76.9 | 5 |
| Protein degradation  (U6) | 10 | 100.0 | 60.0 | 80.0 | 90.0 | 50.0 | 60.0 | 100.0 | 40.0 | 90.0 | 8 |
| Plant defense system  (U7) | 9 | 100.0 | 44.4 | 66.7 | 77.8 | 88.9 | 88.9 | 88.9 | 88.9 | 77.8 | 8 |
| Ubiquitination  (U8) | 8 | 100.0 | 100.0 | 87.5 | 75.0 | 50.0 | 50.0 | 87.5 | 25.0 | 75.0 | 8 |
| TCA cycle  (U9) | 6 | 83.3 | 66.7 | 83.3 | 50.0 | 16.7 | 50.0 | 83.3 | 16.7 | 50.0 | 7 |
| Protein kinases  (U10) | 5 | 100.0 | 20.0 | 80.0 | 100.0 | 80.0 | 100.0 | 100.0 | 80.0 | 100.0 | 8 |
| Mitochondrial ETC/ATP  Synthesis  (U11) | 5 | 100.0 | 80.0 | 60.0 | 40.0 | 40.0 | 60.0 | 100.0 | 40.0 | 80.0 | 6 |
| Starch synthesis & degradation  (U12) | 5 | 100.0 | 60.0 | 100.0 | 80.0 | 100.0 | 80.0 | 100.0 | 100.0 | 100.0 | 9 |
| bZIP TFs  (U13) | 5 | 100.0 | 100.0 | 80.0 | 80.0 | 80.0 | 80.0 | 100.0 | 80.0 | 100.0 | 9 |
