# Supplementary material for: Meta-analysis of drought-tolerant genotypes in Oryza sativa: A network-based approach
Source: PLoS One. 2019 May 6;14(5):e0216068. doi: 10.1371/journal.pone.0216068 (PMC6502313; doi:10.1371/journal.pone.0216068)
Supplement: S13 Table — (DOCX) [file pone.0216068.s013.docx]

**Table S13: Distribution of DEGs in gene clusters shown. All dDTN clusters have ≥ 50% representation from 6 or more data subsets (except 2).**

| **dDTN**  **Clusters** | **No. of DEGs** | **%age of Genes**  **Vegetative Phase - Seedlings** | | | **%age of Genes**  **Vegetative Phase - Leaves** | | | **%age of Genes**  **Reproductive Phase** | | | **Datasets with ≥50% overlap** |
| --- | --- | --- | --- | --- | --- | --- | --- | --- | --- | --- | --- |
|  |  | **GSE41647**  **(DD)** | **E-MEXP-2401**  **(N22)** | **GSE21651**  **(Vandana-only leaf)** | **GSE26280 (DK151-Tillering)** | **GSE24048**  **(Azucena)** | **GSE24048**  **(Bala)** | **GSE26280 (DK151-PE)** | **GSE25176**  **(IRAT109-Flag leaf)** | **GSE26280 (DK151-Booting)** |  |
| Photosynthesis  (D1) | 262 | 94.6 | 62.6 | 86.2 | 63 | 45 | 27.9 | 99.2 | 56.1 | 87 | 7 |
| Cell Wall metabolism  (D2) | 45 | 88.8 | 95.5 | 82.2 | 95.5 | 4.4 | 2.2 | 88.9 | 46.7 | 33.3 | 5 |
| Signaling & PTM  (D3) | 37 | 37.8 | 86.4 | 43.2 | 45.9 | 81.1 | 73 | 89.2 | 89.2 | 45.9 | 5 |
| Ribosome biogenesis  (D4) | 25 | 100.0 | 28.0 | 40.0 | 8.0 | 52.0 | 28.0 | 100.0 | 44.0 | 44.0 | 3 |
| tRNA biogenesis  (D5) | 22 | 81.8 | 72.7 | 50.0 | 18.2 | 63.6 | 36.4 | 77.3 | 59.1 | 41.0 | 6 |
| Development & flowering  (D6) | 11 | 36.4 | 100.0 | 45.4 | 81.8 | 100.0 | 90.9 | 81.8 | 100.0 | 72.7 | 7 |
| Signaling & Transport  (D7) | 8 | 87.5 | 100.0 | 75.0 | 62.5 | 75.0 | 50.0 | 100.0 | 62.5 | 50.0 | 9 |
| α-Linolenic acid metabolism  (D8) | 7 | 71.4 | 85.7 | 57.1 | 42.8 | 57.1 | 57.1 | 100.0 | 100.0 | 71.4 | 8 |
| Glutathione Metabolism  (D9) | 6 | 50.0 | 83.3 | 66.7 | 66.7 | 83.3 | 100.0 | 83.3 | 100.0 | 50.0 | 9 |
| Serine Metabolism  (D10) | 6 | 100.0 | 100.0 | 50.0 | 33.3 | 50.0 | 33.3 | 100.0 | 83.3 | 50.0 | 6 |
| DNA-repair processes  (D11) | 5 | 100.0 | 60.0 | 40.0 | 0.0 | 40.0 | 40.0 | 100.0 | 80.0 | 20.0 | 4 |
| Vitamin B6 metabolism  (D12) | 5 | 60.0 | 60.0 | 20.0 | 40.0 | 80.0 | 60.0 | 100.0 | 80.0 | 100.0 | 6 |
| Cell vesicle transport  (D13) | 5 | 60.0 | 100.0 | 80.0 | 80.0 | 80.0 | 60.0 | 60.0 | 80.0 | 20.0 | 8 |
| Biotic Stress  (D14) | 5 | 40.0 | 80.0 | 40.0 | 40.0 | 60.0 | 80.0 | 60.0 | 80.0 | 60.0 | 6 |
